# Sustained participation in a Payments for Ecosystem Services program reduces deforestation in a Mexican agricultural frontier

**DOI:** 10.1038/s41598-023-49725-7

**Published:** 2023-12-15

**Authors:** Hugo Charoud, Sebastien Costedoat, Santiago Izquierdo-Tort, Lina Moros, Sergio Villamayor-Tomás, Miguel Ángel Castillo-Santiago, Sven Wunder, Esteve Corbera

**Affiliations:** 1 Institute of Environmental Science and Technology, Universitat Autònoma de Barcelona, 08193 Bellaterra, Spain; 2 Conservation International, Arlington, VA USA; 3 Instituto de Investigaciones Económicas, Universidad Nacional Autónoma de México, Circuito Mario de La Cueva Ciudad Universitaria, 04510 Mexico City, Mexico; 4 Universidad de los Andes, School of Management, Calle 21 # 1-20, Bogotá, Colombia; 5 Ostrom Workshop, Indiana University, Bloomington, IN 47408 USA; 6 Departamento de Observación y Estudio de la Tierra, la Atmósfera y el Océano, El Colegio de la Frontera Sur, 29290 San Cristóbal de las Casas, Mexico; 7 European Forest Institute, St. Antoni M. Claret 167, 08025 Barcelona, Spain; 8 Institució Catalana de Recerca i Estudis Avançats (ICREA), Psg. Lluís Companys 23, 08010 Barcelona, Spain; 9 Department of Geography, Universitat Autònoma de Barcelona, 08193 Bellaterra, Spain; 10 Center for International Forestry Research (CIFOR), La Molina, Lima 12, Peru

**Keywords:** Environmental social sciences, Environmental impact, Sustainability

## Abstract

Payments for Ecosystem Services (PES) provide conditional incentives for forest conservation. PES short-term effects on deforestation are well-documented, but we know less about program effectiveness when participation is sustained over time. Here, we assess the impact of consecutive renewals of PES contracts on deforestation and forest degradation in three municipalities of the Selva Lacandona (Chiapas, Mexico). PES reduced deforestation both after a single 5-year contract and after two consecutive contracts, but the impacts are only detectable in higher deforestation-risk parcels. Enrollment duration increases PES impact in these parcels, which suggests a positive cumulative effect over time. These findings suggest that improved spatial targeting and longer-term enrollment are key enabling factors to improve forest conservation outcomes in agricultural frontiers.

## Introduction

Payments for Ecosystem (or “Environmental”) Services (PES) have become a prominent incentive-based environmental intervention since the 1990s^1^. PES schemes often involve short-term contracts (e.g., 2–5 years) with periodic monetary or in-kind payments to landholders. Payments are disbursed in exchange for natural resource management practices, helping to provide key ecosystem services, including carbon sequestration, biodiversity, and water^2^. In the Global South, many PES programs also aim to achieve poverty alleviation and other socio-economic goals^3,4^.

Compared to a counterfactual scenario of no-PES, research has shown that on average PES have induced positive but small impacts on avoided deforestation^5–7^, and that these impacts vary depending on context and implementation features. PES incentives may not always be attractive enough to enroll all forests at risk of deforestation and/or land managers may only enroll the fraction of their property that they are least likely to deforest whilst continuing forest clearing activities in non-enrolled lands (adverse selection bias)^2^. Additionally, PES programs may lack sufficient technical assistance or capacities for compliance enforcement^8^. In some contexts, PES have unintendedly displaced deforestation or forest degradation pressures to non-enrolled lands (leakage)^9^. Modest PES performance often results from programs implemented in “high and far” sites facing low deforestation risk, a low-hanging fruit strategy also across other forest conservation interventions, notably protected areas and community-based forest conservation^10^.

However, PES programs that succeed in targeting sites facing moderate-to-high deforestation pressures have typically had more positive impacts, notably because there is more deforestation to avoid there^11^. Additionally, a few studies have shown that payment schemes can help protect forests even beyond the duration of short-term contracts^12,13^. In a best-case scenario, PES can durably address some drivers of deforestation, e.g., if they build capacities for income diversification away from land-extensive activities, and/or support pro-conservation motivations and social norms beyond the duration of the PES^14–16^. PES can also “delay” deforestation while contracts are implemented, which would still be a positive outcome even if formerly enrolled participants resume foregone deforestation up to baseline speed^17–19^. Obviously, in exceptional cases deforestation rates after PES implementation might also rise above what would have been expected without PES^20^.

**Figure 1:**
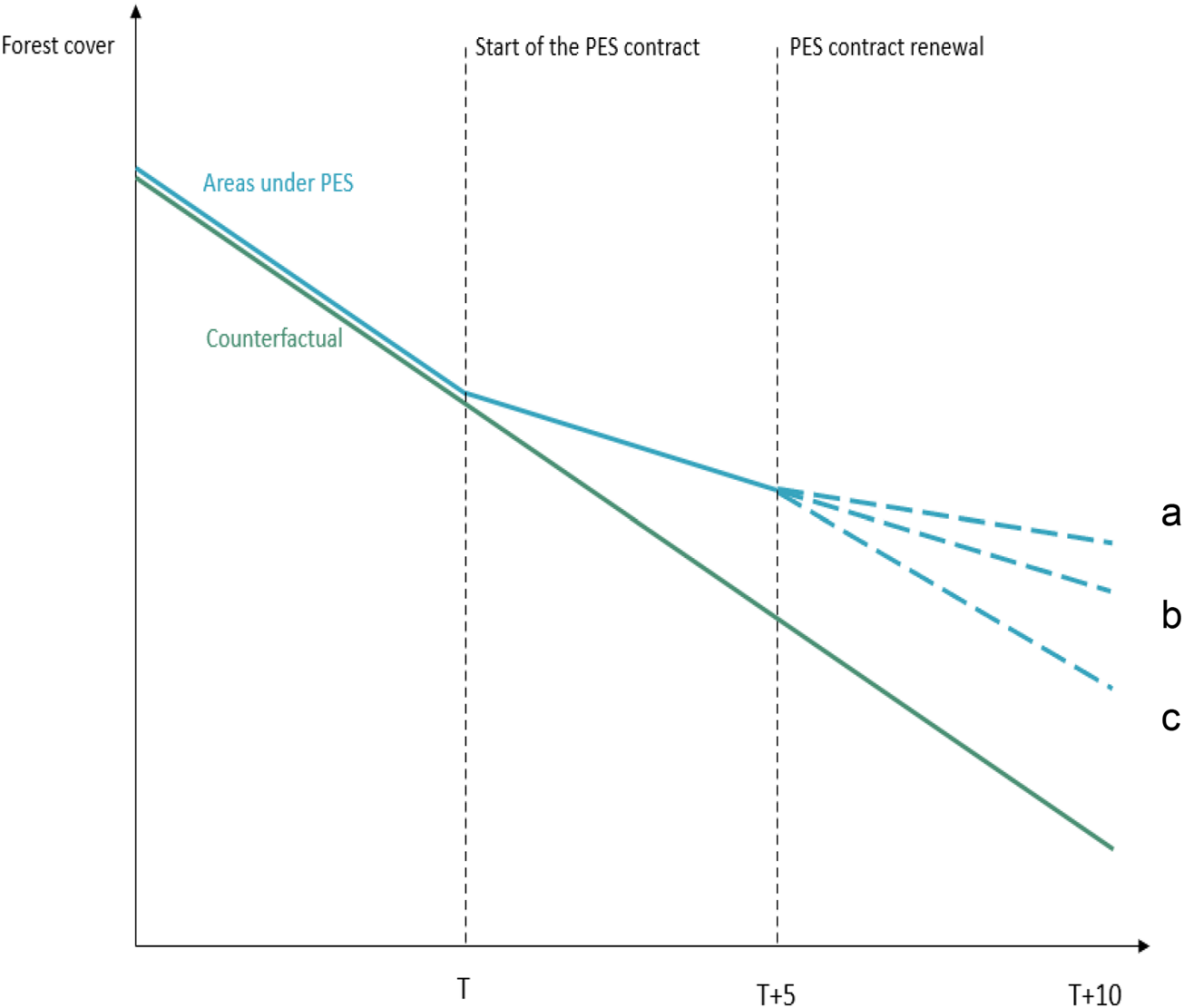
Hypothetical impact of PES programs on forest cover loss associated with contract renewal. Lines a, b and c correspond to three prospective scenarios of forest cover loss. In scenario a, the PES effect increases as the contract is renewed and more forest cover loss is avoided after renewal. In scenario b, the PES effect remains constant over time and PES keep avoiding forest cover loss at the same rate after contract renewal. In scenario c, the PES effect wanes after the renewal and deforestation increases.

PES schemes have generally offered short-term but often renewable contracts, whilst a few schemes have offered longer-term contracts (e.g., more than 10 years or quasi-perpetual contracts), in countries as diverse as Costa Rica, China, and Mexico for two or more decades. However, evidence about the effectiveness of lasting PES schemes over time is scarce^21^. Hypothetically, if well-designed PES programs are implemented in sites that face a high risk of forest cover loss (in the absence of the program), renewable contracts could, compared to the counterfactual scenario of no-PES, induce reinforced cumulative avoided forest loss (Fig. 1a), e.g. if landowners in the second round engage in more lasting, path-defining pro-conservation decisions because their expectations have been changed. Yet, there could also be simply a continuation of the previous impact strength (Fig. 1b). In turn, effectiveness may be diminished if payments become less attractive than agricultural production or other opportunities that turn more profitable or over time, and/or if pre-existing intrinsic conservation motivations are increasingly being crowded out by economic incentives^22^ (Fig. 1c).

In this article, we analyze the contribution of a PES program in avoiding deforestation and forest degradation after 5 and 10 years of contract enrollment. The PES program involves direct payments and technical support for the development of sustainable forest management and conservation plans. We study three municipalities in the Selva Lacandona, in Mexico’s state of Chiapas, an agricultural frontier characterized by high deforestation, collective land tenure, and several PES contract rounds since 2008. We rely on a novel causal inference approach to assess the impacts of the program, combining propensity reweighting and generalized Difference-in-Differences (DiD) models. Propensity scores—the conditional probability of enrolling in the PES given a set of covariates approximating the program targeting criteria—are one way to process the sample selection for “apples-to-apples” comparisons of enrolled with unenrolled sites used as a counterfactual, such that post-reweighting differences in group outcomes are reasonable estimates of PES impact. We disaggregate our findings across municipalities, forest conservation and forest degradation outcomes. Our added attention to forest degradation is important because apparently small disturbances such as selective logging, fires, or poaching^23,24^ can result in large CO_2_ emissions and biodiversity loss, especially in tropical forests, which has often been overlooked^25^.

We confirm findings from previous research conducted in the study area showing an important impact of PES during the first contract period, in sites enrolled since 2008 and 2009^26^. However, we also find that sustained PES participation is associated with cumulative avoided deforestation particularly in high-threat sites: forest cover is 16.5% higher in PES sites enrolled for 10 years, as compared to similar non-enrolled sites. Still, program impacts vary across space and time, which is consistent with the heterogeneity in deforestation risk across the study area.

## Research context and study site

**Figure 2:**
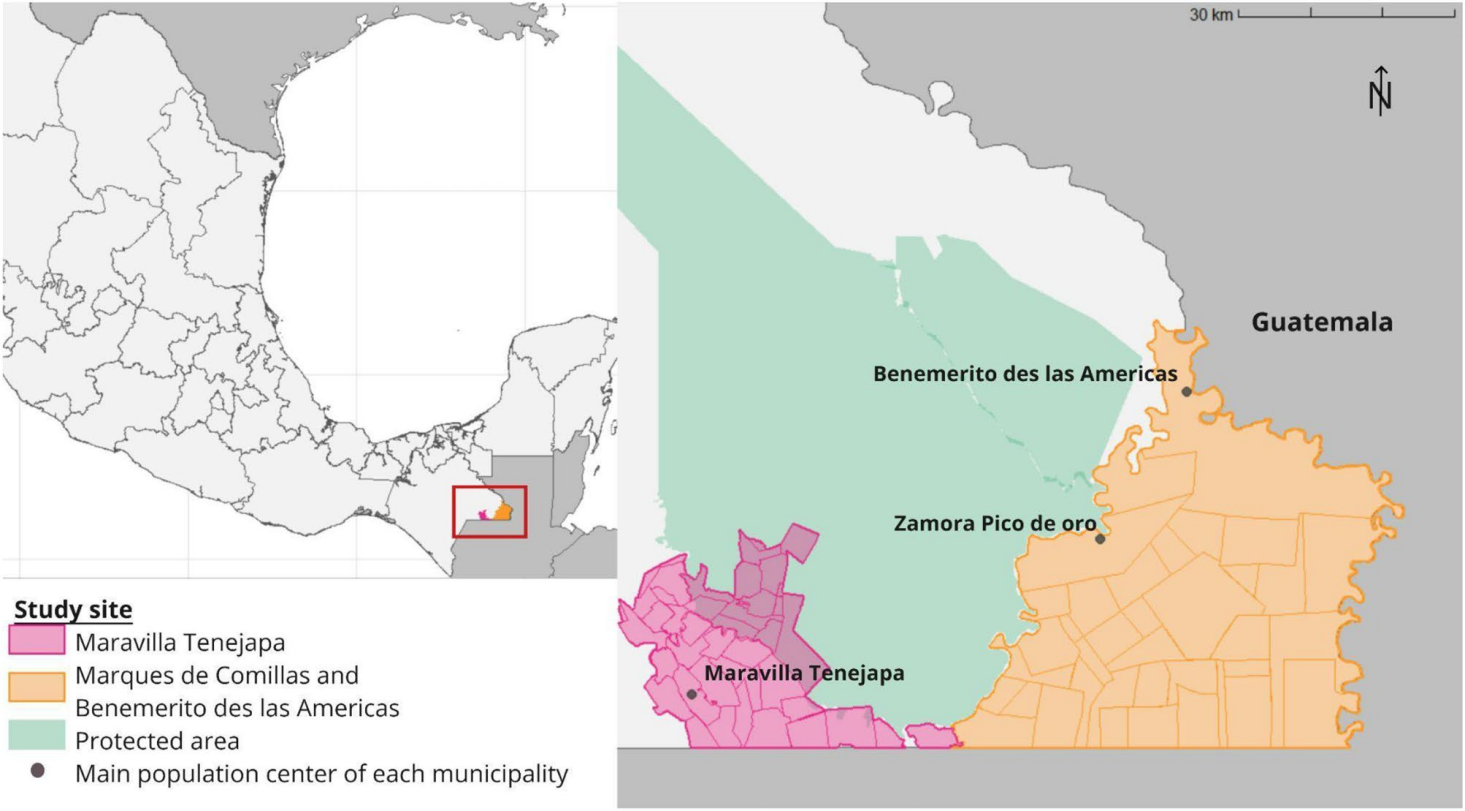
The study site in southeast Chiapas, Mexico. The two study regions (municipality of Maravilla Tenejapa (in pink), and the municipalities of Marqués de Comillas and Benemérito de las Americas (in orange). The inner delimitations of each study area represent the various communities (*ejido*). The Montes Azules Biosphere Reserve and the Lacan-Tun Biosphere Reserve are shown in green. Developed with *R Project for Statistical Computing*, version 4.3.0: https://www.r-project.org/.

Our study area (~ 250,000 hectares) encompasses communities (*ejidos,* a form of collective land tenure in Mexico combining individual and communally managed lands) of three municipalities: Maravilla Tenejapa (MT), Marqués de Comillas (MdC), and Benemérito de las Américas (BdA), southeast Chiapas, Mexico. The municipalities are part of the biological corridor of the Selva Lacandona, a biodiversity hotspot holding as much as 25% of Mexico’s total species diversity^27^. We distinguish two study regions for analytical purposes: MT (Fig. 2, in pink) on the one hand, and MdC and BdA on the other (Fig. 2, in orange), being the latter much flatter and dominated by extensive cattle grazing than MT (Supplementary Table 1).

**Figure 3:**
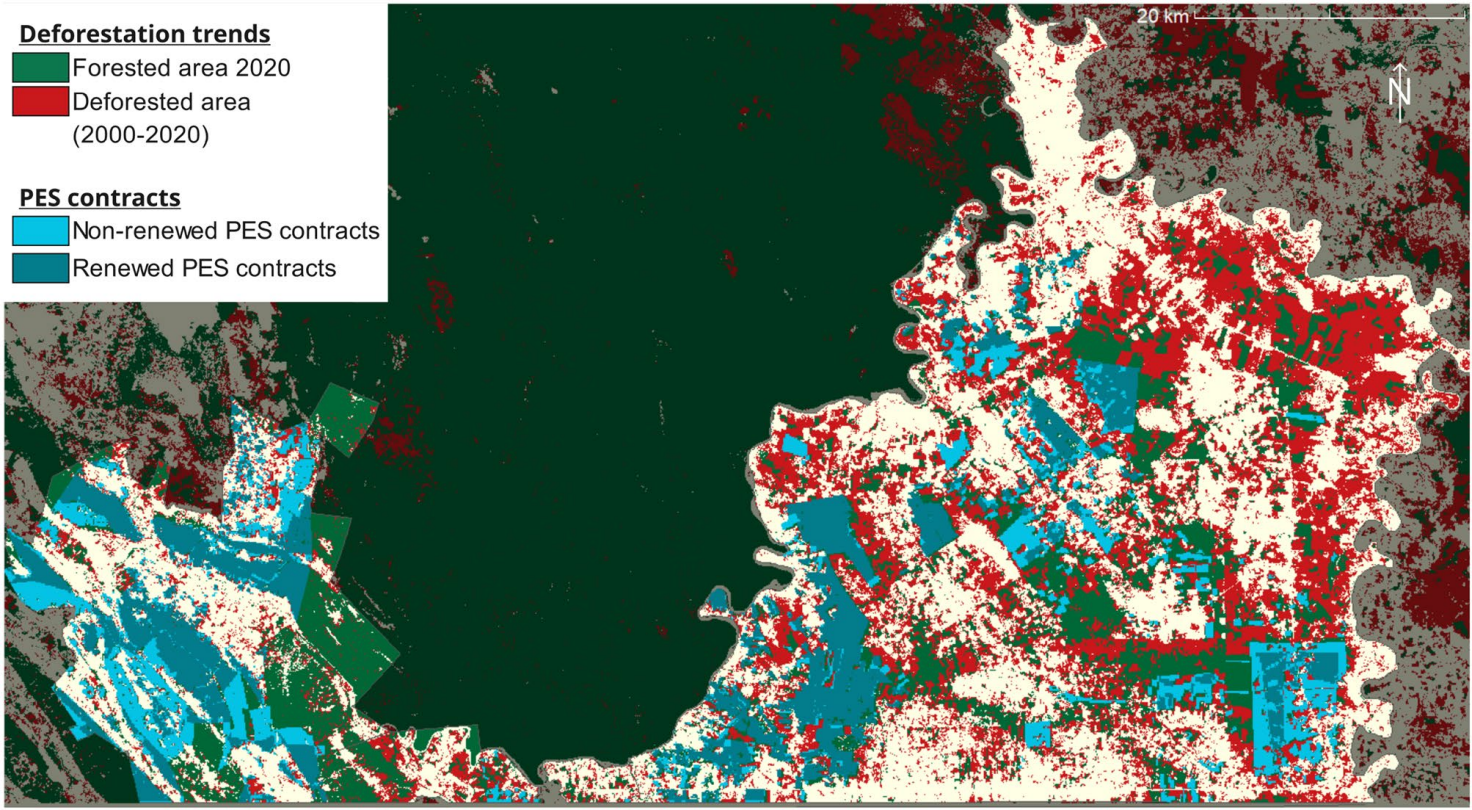
Forest cover change and PES contracts in the study area. Developed with *R Project for Statistical Computing*, version 4.3.0: https://www.r-project.org/.

**Figure 4:**
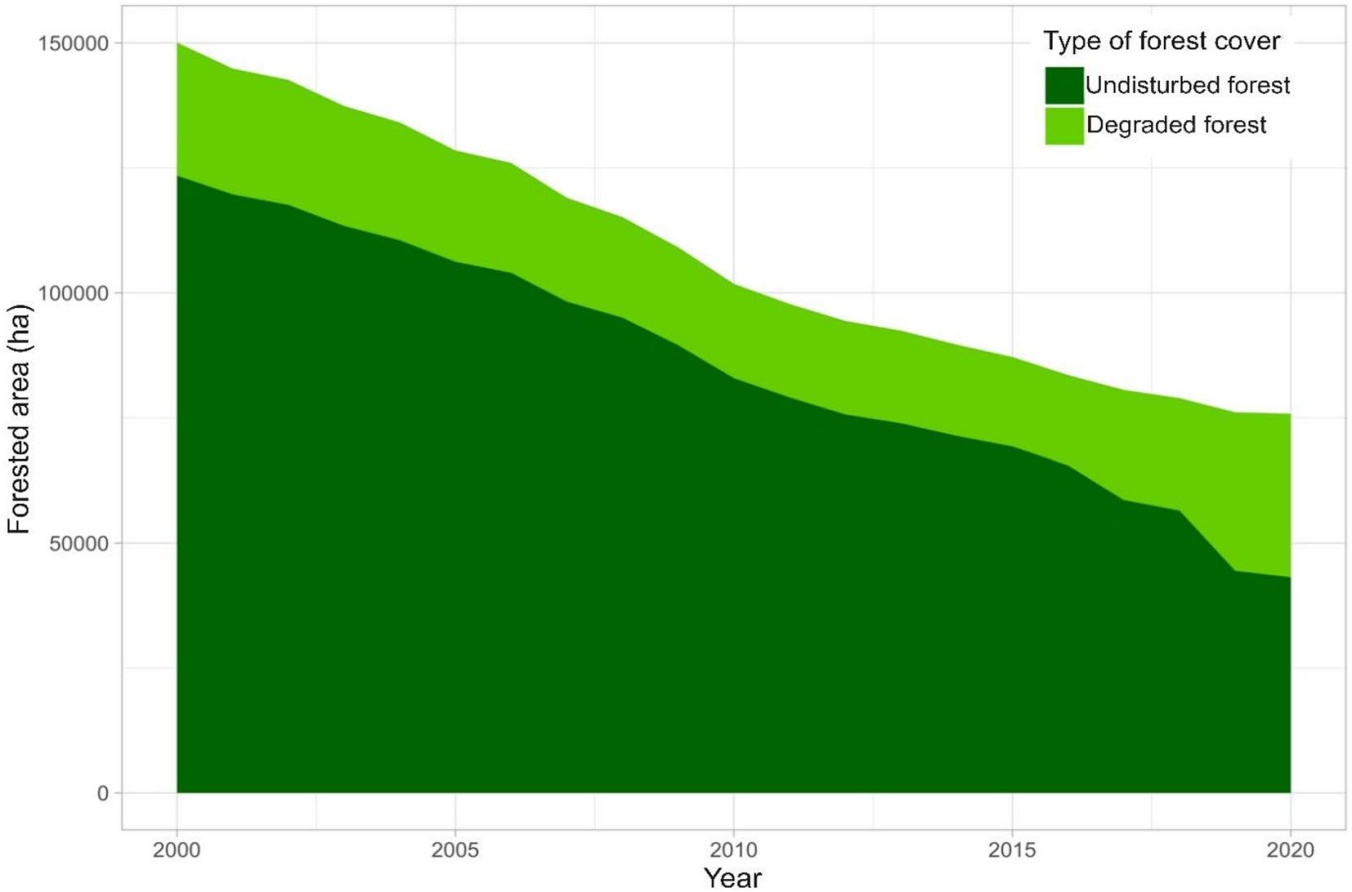
Total annual forest cover and forest degradation between 2000 and 2020 in Maravilla Tenejapa, Marqués de Comillas and Benemérito de las Américas, Chiapas, Mexico (source: JRC database). The figure shows the evolution of forest stock between 2000 and 2020. Half of the total forest cover and undisturbed forest cover has disappeared over the reference period.

In the studied municipalities, half of the 150,000 hectares of standing forests in 2000 were converted to other uses by 2020, whilst forest degradation also increased after 2015 (Figs. 3 and 4, and Supp. Fig. 1). Such a degree of forest loss, with annual deforestation rates of about 1.5–3% between 2000 and 2020, represents one of the highest in the country. The flat topology in MdC and BdA has favored the expansion of livestock, agriculture, and plantations of oil palm and rubber trees—the more fertile soils near the rivers bordering the region being at highest risk^28^.

Despite some changes in rules, most PES operations have followed a similar approach: 5-year renewable contracts at 550–1000 MXN (31–57 USD) per hectare, conditional on forest conservation and compliance with a forest management plan. PES applicants can enroll either individual or communally managed forests, the latter being endorsed by the community assembly. CONAFOR administers the programs, and monitors compliance through satellite imagery and random field visits. Applicants’ selection, the design of forest management plans, and technical assistance have been performed by tertiary non-governmental organizations or forestry consultants. Officially, contract incompliance should lead to contract interruptions. In practice, when non-compliance is detected, contracts can eventually be renegotiated, e.g., changing project areas or reducing payments.

Two national PES programs abbreviated PSA-H (supporting forests for watershed protection) and PSA-CABSA (targeting carbon sequestration, biodiversity conservation and agroforestry) were launched by the Mexican National Forest Commission (CONAFOR) in 2003 and 2004, respectively^29,30^. In 2008, these programs were merged into the broader forest conservation strategy known as *ProArbol*, which was renamed as PRONAFOR in 2013. In the study area, the first PES contracts were signed in 2008. In 2010, the region also became a target area for the country’s REDD + early-action program (*Programa Especial Selva Lacandona*—PESL), offering higher per-hectare conservation payments than pre-existing PES schemes (1000 Mexican Pesos (Mxn) versus Mxn 550 per hectare). Consequently, after 2011, PESL rapidly became the main source of PES funding in the region (Supplementary Figs. 2 and 3). Between 2008 and 2018, 41 out of the 71 communities in the three municipalities studied had enrolled some or all their forests into PES programs, reaching a total of 49,200 hectares.

PES contracts have been renewed in 31 out of the 41 participant communities in the study area. In hectares, renewed contracts represent 60% of the total historically enrolled area (Supplementary Fig. 3). Interviews with several local community members suggest that non-renewal is due to discontinued land-manager participation, but a few cases of involuntary non-renewal have also taken place (e.g., applications rejected due to missing paperwork, funding shortages, or changed eligibility criteria). Among the forest enrolled in only one contract, 25% corresponds to non-renewed contracts, either because participants were not eligible anymore under program rules, decided to withdraw, or their application for funding renewal was not successful. Finally, 8 communities enrolled forests in PES after 2013, and we do not have information on eventual renewals after 2018.

A previous impact evaluation found that in communities of MdC and BdA that joined PES in 2008 and 2009, forest cover in enrolled parcels after 5 years of PES was 9–11% higher than in similar control plots^26^. Below we extend this evaluation in terms of temporal and spatial scope (Fig. 3). We consider all the PES contracts initiated or renewed between 2008 and 2018, and add the communities of MT. Our analysis also includes forest degradation outcomes and is thus more comprehensive while also disaggregating the analysis to explore spatial and temporal heterogeneities.

## Results

### Effect of single contracts are heterogeneous due to differences in deforestation risk

We first focus on the effect of PES after a single contract. We distinguish two subgroups of PES parcels: (i) those under contracts that have been renewed after 5 years (i.e., we look at the effect at t + 5, before renewal, of the sample presented in the previous section), and (ii) those enrolled early in the program which did not renew after 5 years, also including those that were too recent to be renewed in our sample because they enrolled after 2013.

**Figure 5:**
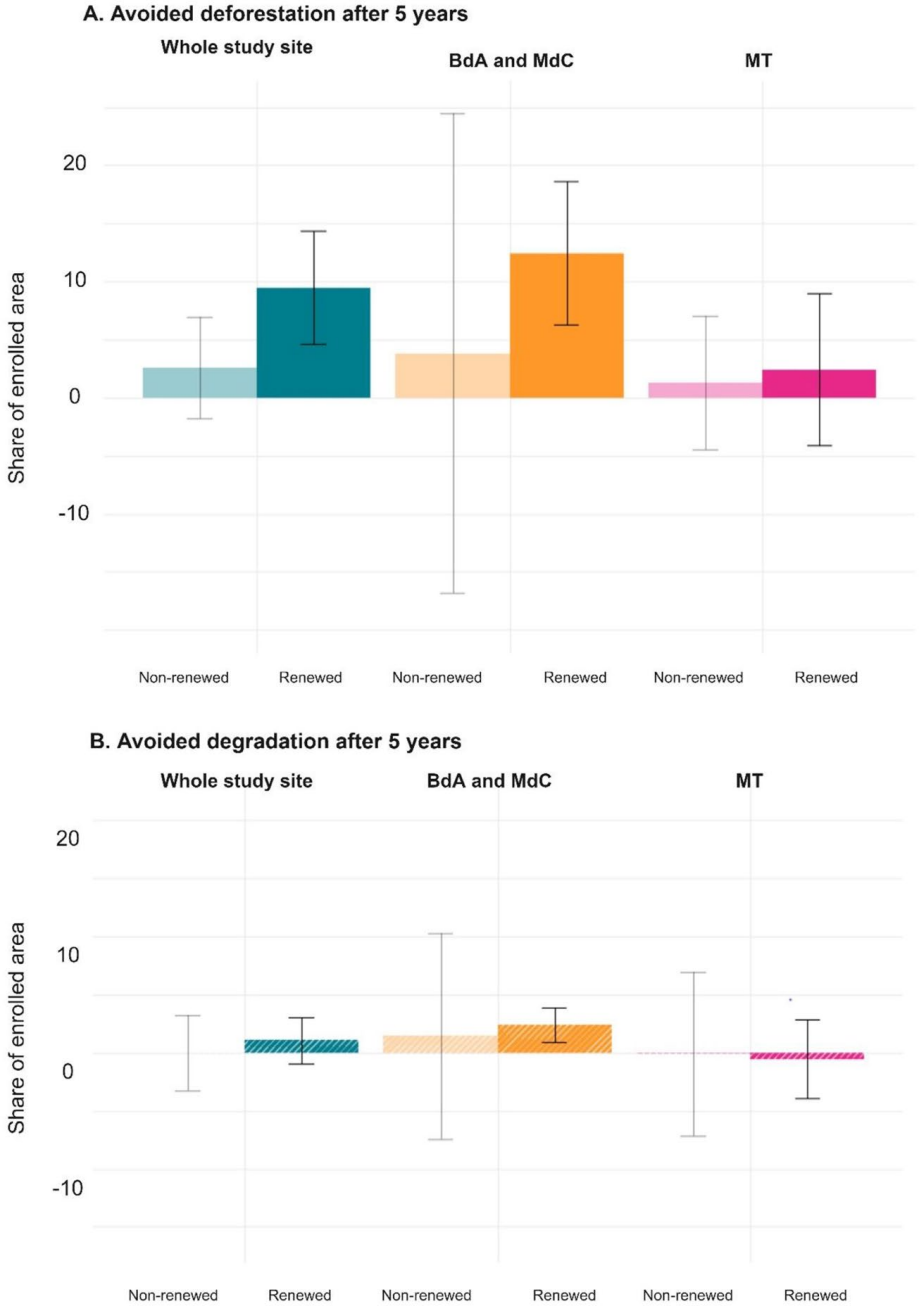
Effect of non-renewed and renewed programs after a single (5-year) contract. We compare the effect of renewed and non-renewed contracts, the left light-colored bar representing the latter and the right, darker one the former. The top-panel shows avoided deforestation, the bottom one avoided degradation (hatched). Error bars represent 95% confidence intervals.

In forest parcels where the initial 5-year contract has been renewed, the impact of PES on deforestation is significant after 5 years (Fig. 5; Average Treatment Effect on the Treated (ATT) = 9.5%, 95% CI [4.6%; 14%]). For parcels only enrolled once, deforestation reduction is statistically non-significant (ATT = 2.6%, 95% CI [− 1.8%, 7%]). Further comparisons among subgroups all result in insignificant differences, probably also due to the small sample size (Supplementary Fig. 7 and Supplementary Note 1).

The two subgroups of PES parcels are quite similar on average in terms of elevation, slope, and distance to roads, but the sample of renewed contracts has more parcels with higher levels of forest cover in 2007, which are also slightly closer to rivers, to municipal capitals, and to protected areas, but had relatively fewer night-time lights in 2014—as compared to the parcels that only once enrolled in PES (Table 1). This suggests, on the one hand, that renewed contracts were on average located in areas with relatively higher forest cover but closer to rivers, and therefore subject to a higher deforestation risk due to expectations that the soil near rivers is more fertile for crops. On the other hand, it also suggests that there may be other reasons explaining the lower level of forest cover in 2007 of those plots only enrolled once, including non-observable factors or program design challenges. In this regard, when processing data, we found that some of these early participants had their PES plots’ boundaries irregularly drawn, covering poorly forested areas. We thus suspect that CONAFOR redrew the boundaries of affected plots at t + 5 and, in some cases, cancel renewal if the original level of forest cover was already insufficient to justify participation.

**Table 1:** Observable characteristics of the subgroups by contract renewal status.

	Renewed (N = 28,838 pixels)	Non-renewed (N = 20,611 pixels)
Elevation (m)		
Mean	295.00	271.86
SD	161.59	153.89
Range	111.41–899.19	114.16–913.99
Slope (°)		
Mean	11.85	10.47
SD	8.88	7.88
Range	1.48–59.23	1.59–59.50
Forest cover in 2007		
Mean	0.94	0.85
SD	0.17	0.26
Range	0.00–1.00	0.00–1.00
Distance to inland water (m) (*)		
Mean	2670.37	3004.70
SD	1836.63	2317.98
Range	12.24–10,513.43	9.65–10,393.24
Distance to roads (m)		
Mean	1655.84	1625.91
SD	1251.62	1272.94
Range	21.07–6310.31	14.03–6554.33
Distance to main city of the municipality (*)		
Mean	15,243.17	17,542.41
SD	8219.64	11,277.21
Range	677.40–37,534.88	267.53–40,992.30
Distance to protected area (m) (*)		
Mean	7490.04	13,648.94
SD	7576.73	12,246.90
Range	0.00–37,409.02	0.00–40,841.45
Lights at night (2014) (lux)		
Mean	4.29	4.67
SD	2.76	2.77
Range	0.00–8.00	0.00–8.00
Demographic growth (ejido level)		
Mean	0.12	0.21
SD	0.24	0.25
Range	− 0.33 to 1.00	− 0.33 to 1.00

SD = Standard Deviation. An asterisk (*) identifies the variables for which the values are statistically different between the two groups at the 10% threshold (t-test).

As for heterogeneous effects across subregions, we find that PES impacts on avoided deforestation are only statistically significant in MdC and BdA for parcels where contracts have been later renewed. We explain this regional heterogeneity again due to the fact that the deforestation risk is expected to be lower in MT because it is an area where there is less risk of agricultural conversion due to the rugged topography (Supplementary Tables 1 and 2): enrolled parcels in MdC and BdA are located on average on land that is less elevated, less sloped, with more forest in 2007, less deforested in the past (2000–2008), and closer to rivers.

### Renewed PES contracts implemented in at-risk parcels effectively reduce deforestation

We now focus on parcels where PES contracts have been renewed at least once, i.e., they have been in the program for 10 years or more. In the 28,800 hectares enrolled in renewed contracts, average yearly deforestation is 0.54%, compared to the ten times higher loss of 5.5% in (matching) control areas (see Supplementary Figs. 4 and 5). However, to attribute any effect to the PES program, we need to compare enrolled areas to non-enrolled parcels that are observationally similar for the entire period of study. For this purpose, we use a general DiD estimator that labels the year before the first year of contract as baseline and the tenth year of contract implementation as endline (see “Methods” below).

**Figure 6:**
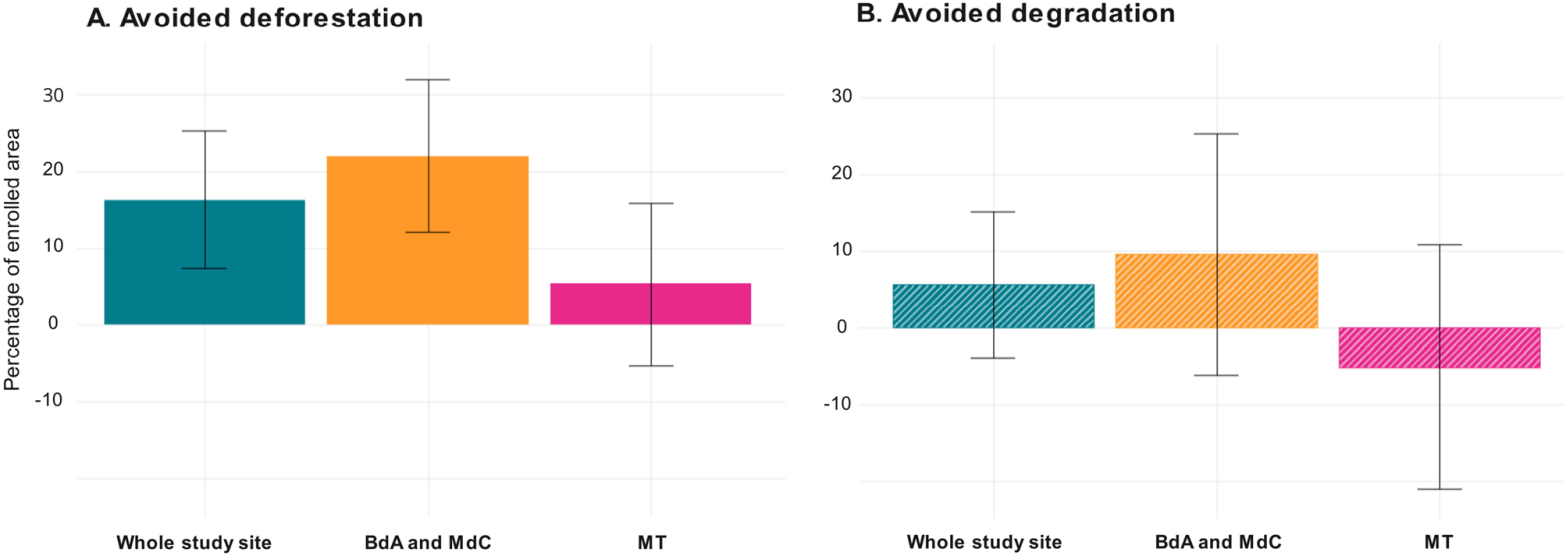
Cumulative 10-year effect of two consecutive PES contracts. Cumulative average effect of 10-year PES contracts in the whole aggregated study sample (combining the two regions), and then distinguishing across the two selected regions, expressed as percentage of enrolled area. The left panel indicates avoided deforestation (difference in forest cover per pixel in enrolled parcels versus similar non-enrolled forest parcels). The right panel (hatched) shows avoided degradation (difference between intact forest cover per pixel in enrolled versus non-enrolled forest parcels). The PES effect on avoided deforestation is statistically significant (95% CI) in the aggregated study sample, and it is driven by the significant effect found in MdC and BdA; the effect on avoided degradation is insignificant.

**Figure 7:**
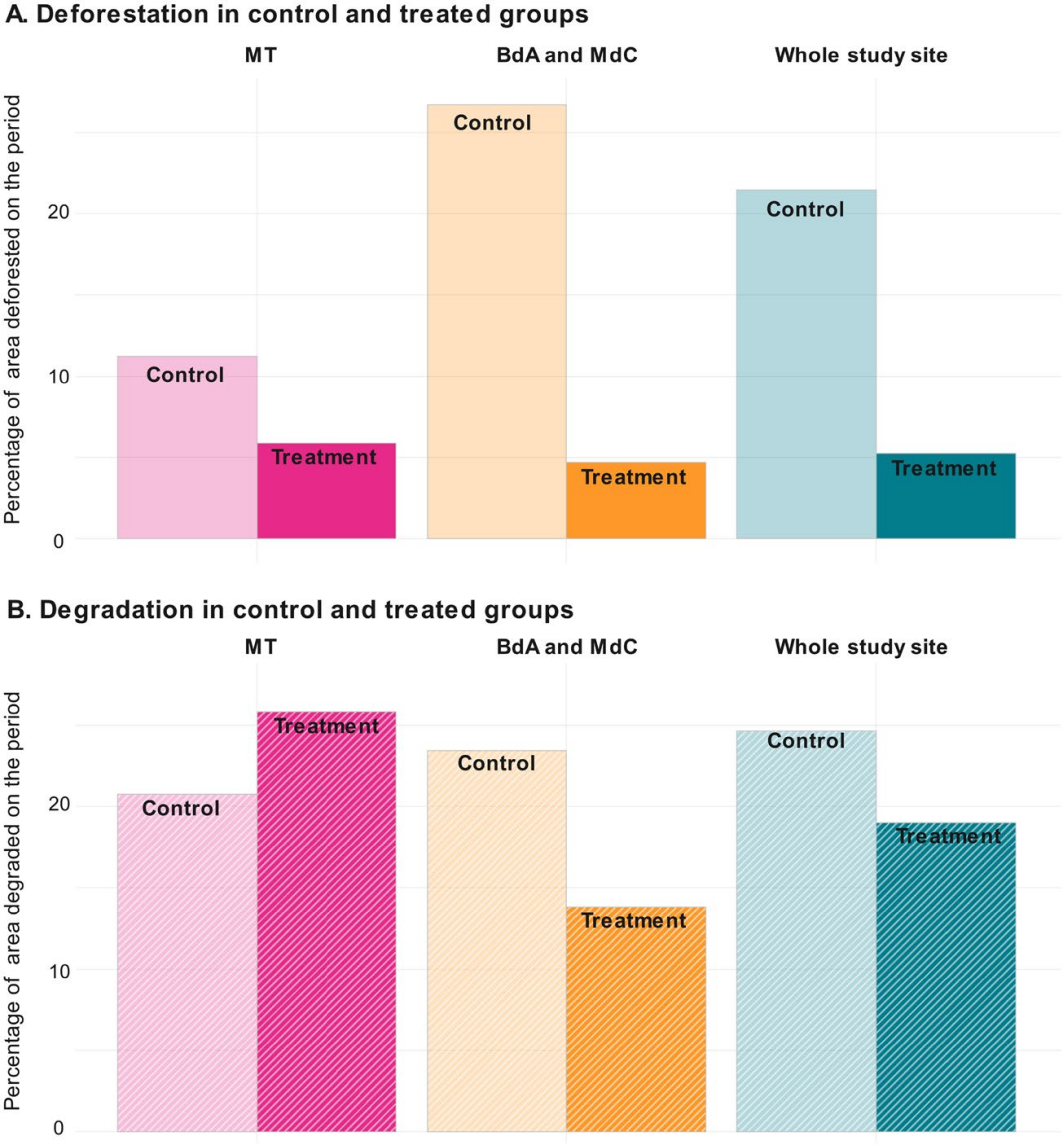
Difference in deforestation trends between treated and control parcels across the study region. Panel (**A**) shows the level of deforestation in treatment and control groups during PES, while Panel (**B**) shows the level of forest degradation. Deforestation and forest degradation are expressed as share of the area.

Overall, forest cover is on average 16.5% higher (95% CI [5%; 27%]) in 10-year PES-enrolled parcels, as compared to non-enrolled comparable parcels (Fig. 6A). We estimate that between 2008 and 2020, PES have avoided the deforestation of *circa* 4700 ha across the three studied municipalities (95% CI [1440; 7790]). If we look at trends in deforestation and degradation across treated versus non-treated parcels, we can observe that the former were almost three times larger across the three municipalities, and particularly more significant in MdC and BdA (Fig. 7A). As regards avoided degradation, the difference in trends across the whole study region is almost the same. Surprisingly, we also observe that forest degradation in treated parcels in MT is slightly higher (although not statistically significantly) than in non-treated ones (Fig. 7B).

**Figure 8:**
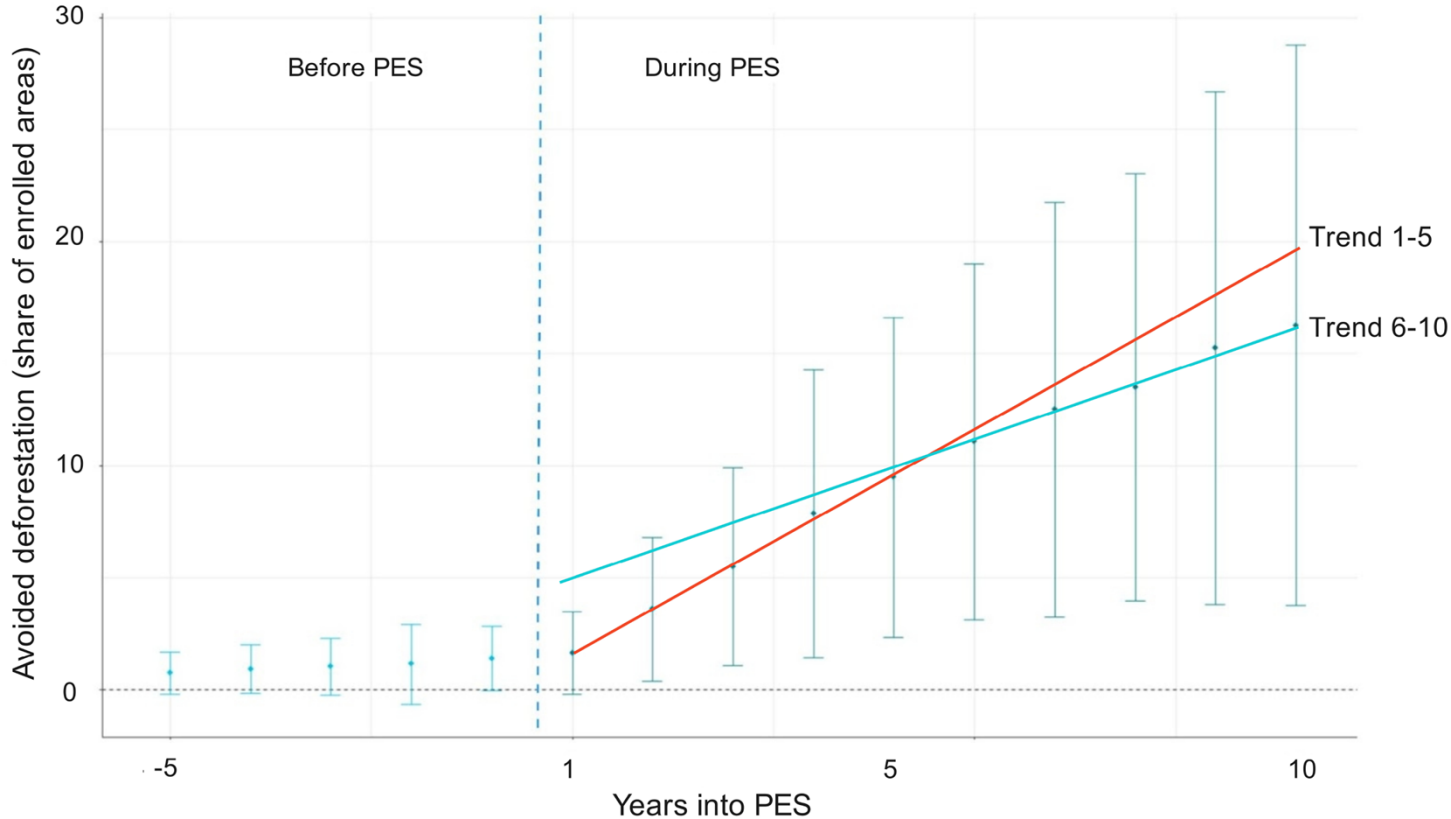
Event-study graph of the annual effect of PES in parcels under renewed contracts. The graph shows the program’s avoided deforestation effect over time in parcels enrolled for 10 years or more. Avoided deforestation, represented on the y-axis, is measured as the share of enrolled areas that would have been deforested in the absence of treatment. Time, represented on the x-axis, represents the number of years after entering PES. For instance, 5 years after the beginning of the contract, PES had on average avoided 9.5% (95% CI [4.6%; 14%]) of cumulative deforestation. The trend lines capture the annual conservation effect of PES and show that such effect is weaker after the first contract renewal.

Figure 8 shows the average effect on forest cover by year, both before and after the first and subsequent years of PES enrollment. Results suggest that avoided deforestation on average increased as a function of years of enrollment, albeit the annual effect of PES on avoided deforestation slightly decreased during the second enrollment period. The standard error increases simultaneously, signaling important uncertainty around the average impacts, though not enough to render the results statistically insignificant. Overall, we can cautiously say that the longer the parcels are enrolled in PES, the more deforestation PES was avoided: the program induced a cumulative avoided forest cover loss over the implementation period, at least in the subgroup of parcels that have been enrolled at least 10 years, with a potentially decreasing conservation effect over the years.

Regarding leakage, we did not find evidence of an increase of deforestation in unenrolled forests within participant *ejidos* (using matched enrolled forests in participant *ejidos* as counterfactuals), suggesting that PES have not caused statistically detectable intra-*ejidos* leakage from enrolled to non-enrolled forests (see Supplementary Fig. 6).

The analysis of the two distinct regions (MT vs. MdC and BdA) reveals again important differences. The long-term effect of the program in MT is more than four times lower than in MdC and BdA, and statistically insignificant (Fig. 5). In BdA and MdC, a 10-year PES contract avoided the deforestation of 22% in the enrolled areas of these two municipalities. Out of the total 4700 ha of estimated avoided forest loss, 3700 ha are in MdC and BdA (95% CI [830; 5800]). This can be explained again by the fact that the two study regions have very distinct deforestation risk patterns.

### No significant PES effect on forest degradation

Running the same analysis with forest degradation as outcome variable, our findings do not indicate a significant effect of PES on avoiding degradation over a 10-year period (Fig. 6B). Twenty percent of forests enrolled that were undisturbed at the beginning of the PES contract became degraded during implementation, which suggests that a portion of PES participants probably practiced selective, low-intensity logging in enrolled areas. This is consistent with previous research in these two municipalities^26^. However, PES reduced degradation on parcels in MdC and BdA during the first contract (Fig. 5B, MdC and BdA for renewed contracts), thus indicating a short-term effect which weakened over the second contract period (Fig. 6B, MdC and BdA), and which in other studies has been referred to as the “honeymoon effect”^31^. This may also indicate that PES participants realized that CONAFOR tolerated selective logging in PES parcels, but more research is needed to corroborate this hypothesis.

### Assessing the robustness of the findings

**Figure 9:**
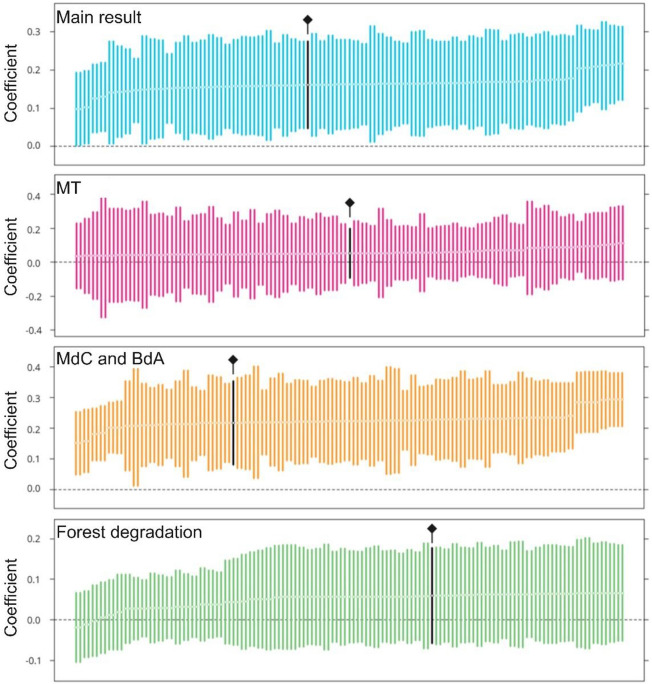
Specification charts for the robustness of our main findings. For each estimate, we tested 132 alternative models of the estimation of the cumulative 10-year effect of two consecutive PES contracts on avoiding deforestation or forest degradation (see “Methods”). The first panel shows the final estimate and the other 132 estimates of the treatment effect in terms of deforestation. The two following panels show the different estimates of the effect of PES in each region (MT on the one hand, and MdC with BdC on the other). Finally, the last panel shows the various estimates of the PES effect in terms of avoided forest degradation in the whole study region. The estimates we used in our results section are highlighted in black.

The validity of any impact evaluation relies on the credibility of the assumptions used to identify the counterfactuals. To ensure that our main estimates on the cumulative 10-year effect of two consecutive PES contracts are not biased by arbitrary model specifications, we tested 132 alternative models corresponding to variations in the choice of matching variables (see “Methods”). Figure 9 shows that our results from Fig. 6 remain stable *vis-a-vis* most changes in specifications: for the main results, and particularly in MdC and BdA, all models indicate a significant effect, with only moderate variations in the magnitude of the main effect. Finally, for forest degradation, none of the alternative models suggest a significant treatment effect, and the sign of the main effect varies across specifications, confirming again that we cannot reject the null hypothesis of no PES impact on forest degradation.

Another robustness check consists in testing the credibility of the assumption that in the absence of treatment, the outcomes in the treated group and control group would have varied following a parallel trend (see “Methods”). To do so, we tested the credibility of this assumption by assessing the sensitivity of our findings to different differences in pre-PES trends between treated and control groups. We confirmed the credibility of the parallel trend assumption, and therefore the validity of our main findings. Furthermore, out of the 132 alternative models tested as robustness checks, 81% are robust to a deviation greater than the largest deviation observed in the pre-treatment trends (see Supplementary Figs. 9–12).

## Discussion

We have analyzed the medium-term impacts of PES in avoiding deforestation and degradation in an agricultural frontier in Chiapas (Mexico), both after a single PES contract and after contract renewal. Using a novel counterfactual approach, we have shown that PES contracts avoided deforestation under a single, and under renewed PES contracts but at a lower rate, at least in one region of our study area. We have found that forest cover is about 16.5% higher in sites enrolled at least twice in PES contracts, as compared to never enrolled sites. This represents a cumulative total of about 4700 ha of avoided deforestation between 2008 and 2020 in the three municipalities studied. These findings are consistent with a previous, yet less exhaustive impact evaluation and echo qualitative observations in six communities of the study region^26,32^. However, we did not find a sustained impact of the program on forest degradation.

We have also demonstrated that PES impacts were not only sizable, but also increased over time on lands where PES contracts had been renewed throughout our study site. Additionality has been important in sites enrolled in renewed contracts, which have been effective in avoiding deforestation since the first year of enrollment. Renewed PES contracts targeted more forests facing high deforestation risks over time because CONAFOR increasingly added deforestation risk into their selection criteria^22,29^. Sustained PES contracts can thus induce cumulative impacts of avoided deforestation over time if programs keep targeting plots genuinely at risk of land conversion but also if contractual incentives are adapted to motivate landholders to continue enrolled and compliant over time. Achieving the latter requires payments to remain competitive *vis-a-vis* conservation opportunity costs. For example, by increasing per-hectare payments from MXN 550 (USD 31) to MXN 1000 (USD 56) from 2010 onwards, the PESL program most likely motivated more landholders to enroll in PES, and previous participants to enroll more forests with higher opportunity costs in subsequent contracts. After 2014, however, land enrollment in the study region declined (see Supplementary Fig. 12) possibly because the program’s budget declined and payments per hectare had been kept constant at MXN 1000 since 2010 whilst cattle and agricultural profitability increased. Hence, the observed cumulative gains in terms of avoided deforestation may dissipate if PES do not adapt to the profitability dynamics of alternative land uses, and if they do not reinforce other non-monetary or intrinsic motivations that may underpin participation in conservation programs.

We have also shown that PES impacts on avoided deforestation vary much across space and time, which is consistent with previous findings^20,33^. Some of these variations corroborate well-known PES design features for effectiveness, such as targeting the enrolment of parcels facing moderate-to-high opportunity costs^3^. Program design and implementation features should also remain adaptive to changing local conditions and expectations among participants (e.g., land tenure, shifting capital and labor constraints, demographic changes, contradictory effects of other land-use incentives favoring extensive practices), if they are to boost long-term conservation efforts^34,35^.

Although we did not evaluate intra-*ejido* leakage (i.e., from PES-enrolled to non-PES forests within the same *ejido*), we theoretically expect two types of potential leakage occurring in the study area: first, PES enrollment could enable participants to directly acquire and subsequently deforest lands in neighboring communities; second, PES participation in the study region or elsewhere could induce additional deforestation through its influence on equilibrium prices for land and agricultural products^9^. We believe the first type of leakage is negligible because landholders in the study area rarely hold lands in communities outside of their *ejido* of residence, since land tenure typically entails additional responsibilities such as days of unpaid labor and periodic involvement in community assemblies and decision-making processes^32,35^. Yet, we acknowledge that the second type of leakage is indeed possible because of the large amount of land enrollment in PES in the study area and in other neighboring regions. Future studies could explore the presence and magnitude of both intra- and inter-community leakage induced by PES in implementation contexts.

Additionally, as with any observational study, we acknowledge there might be unaccounted confounding variables that could influence the results. These may include, for example, unobservable characteristics of PES participants, such as their level of social capital or collective action, or the effect that tertiary organizations and forestry consultants may exert on the quality of technical assistance and monitoring frequency and, subsequently, on compliance (see “Limitations” in “Methods”). Notwithstanding, our research shows the potential of combining direct payments with the development and implementation of forest management plans to boost rural communities’ conservation efforts, and indirectly contributes to studies on the ecological benefits of community-based forest management and Indigenous Peoples’ tenure regimes^36,37^. Furthermore, the fact that the studied PES program does not supersede existing local tenure institutions, has a voluntary character, and aligns well with those households whose will is to protect at least some of their standing forests explains its relative success in avoiding deforestation^38^. Future studies could examine the individual contribution of the various components of a PES scheme through, for example, randomized control trials with different treatment arms (e.g., with or without technical assistance, or with distinct monitoring intensities by tertiary organizations), and could also explore how PES enrollment and performance may be mediated by local environmental motivations.

To conclude, we have contributed to the growing body of literature on the impacts of PES as a streamlined, dynamically applied conservation instrument. Our results confirm that sustained and renewed PES contracts can effectively incentivize communities to improve conservation outcomes in areas where forests are at risk of being deforested. Programs supported by a long-term strategy and relying on adaptive management processes can ensure that incentives remain aligned with the type and magnitude of land conversion pressures, and they can also support changes in community rules, norms and decision-making processes that aim to foster biodiversity conservation.

## Methods

We used a quasi-experimental counterfactual-based research design comparing change of forest cover and forest degradation between enrolled sites and similar but non-enrolled sites, before and after the beginning of PES.

### Choice of the unit of analysis

We ran the analysis at the grid cell level. Grid cells are adjacent spatial squares that are used to characterize changes in forest cover and degradation, as well as other spatially explicit characteristics overlapping the cells. Grid cells approximately catch sub-community household decision-making regarding land use change, although there are not perfect substitutes for field-based data collection at plot level^39^. Given that forest cover change is often the consequence of small-scale land-use changes to agriculture^40^ and that the mean agricultural plot size in the study area is around one hectare, we used a one-hectare grid cell as a unit of analysis. For our main results, we only kept the pixels that had at least 10% of their surface covered by forests in 2007. However, other thresholds were tested as part of our robustness checks (see Supplementary Figs. 8–11).

### Enrolled and unenrolled cells

We used the PES contract polygons enrolled from 2008 to 2018 provided by CONAFOR to identify enrolled sites. We performed minor corrections to ensure that each PES polygon is within the limits of the community receiving the PES contract (see Supplementary Note 2). Every pixel with its centroid situated inside a PES contract was identified as enrolled. In the non-enrolled control group, we included cells located in communities never receiving PES. As our data considers temporal variations, we also labeled as non-enrolled the grid cells corresponding to cells that will participate in the treatment, but have not participated yet (e.g., if a cell overlaps with a contract starting in 2010, it was labeled as non-enrolled before 2010, and as enrolled in 2010 and each subsequent year). In the robustness checks (see Supplementary Figs. 8–11), we tested whether excluding these eventually enrolled cells from the control group affected our main findings. Finally, we excluded cells located in communities receiving PES but located outside the limits of PES contract polygons, as we suspect that these cells are more likely to be deforested either because they were not included in PES as their owners had intentions to deforest these plots, or because of eventual leakages occurring from PES to non-PES forests within a participating community. Such cells were used to test for on-site leakage (see Supplementary Note 3).

### Outcome variables

We used the European Commission’s Joint Research Centre tropical moist forests (TMF) database (1990–2020) to create our outcome variables^41^. This database offers worldwide yearly data on deforestation and degradation. Deforestation is defined as long term total clearing of the pixel, whereas degradation is defined as a short-term disturbance of the pixel (see Supplementary Note 4). We reclassified their nomenclature to exclude forest regrowth. This ensured that tree plantations—which are often classified as forest regrowth in land-use analyses—were not considered as forest cover in this research (see Supplementary Note 4). While measuring the impacts of PES on forest gain is of interest, we consider we do not have accurate data sources to estimate this impact, and therefore focus our analysis on avoided deforestation and avoided degradation. We used the share of total forest cover in each cell to quantify yearly forest cover, while we used the accumulated sum of degraded forest cover in each cell to quantify yearly degraded forest.

### Confounding variables

Our identification strategy assumes that, in the absence of PES, “observationally similar” enrolled and non-enrolled cells (used as a counterfactual) would have had the same deforestation and degradation outcomes. We used an Inverse Probability Weighting based on the Propensity Score to ensure that, after matching, our samples of enrolled and unenrolled cells have the same probability to be included in the PES program. To select the set of confounding factors, we scrutinized multiple variables that could potentially affect both the probability to enroll in PES and the level of deforestation. This included slope, elevation, past deforestation trends (share of grid cell forested before entering PES), distance to rivers, distance to road, distance to municipal capital, distance to the nearest protected area, night-time lights, and demographic change between 2000 and 2010 (in %). Spatial lags (computed using queen-contiguity spatial weights) on geophysical variables were also added to consider spatial dependency, i.e., the fact that characteristics in neighbor cells may influence values in a cell. All the confounders were at the pixel level except for demographic growth that was obtained from census data at community level.

### Identification strategy

We used a generalized Difference-in-Differences (DiD) estimation with multiple time periods to study the effect of PES on deforestation and degradation. Our estimations of effects rely on doubly robust estimators, i.e., we combine a DiD design with the Inverse Propensity Score, with reweighting based on the Propensity Score explained in the previous section. This estimator is less likely to be biased than either classical DiD or matching procedures because the doubly robust property ensures that models are correctly specified even if either the DiD outcome regression or the Propensity Score reweighting is mis-specified^42,43^. Furthermore, the generalized DiD estimation with multiple time periods allows to compute event-study estimates, i.e., summarizing how the effect varies as a function of different lengths of exposure to PES, notably taking account that not all PES contracts have initiated the same year. Our main estimate is extracted from these event-study regression: we mainly focused on the ATT in each cell at t + 9, which represents the effect of PES 10 years after entering the program, as well as effect at t + 4, which represents the effect 5 years after entering the program.

### Robustness to model specifications

Propensity score models require various assumptions and choices to be made, while small variations in the choice of variables can lead to substantial differences in the sign and magnitude of impacts^44,45^. To ensure our results were not the result of an arbitrary choice, we tested the robustness of results in 132 different models, corresponding to different sets of confounding covariates and other parameters. In the alternative models we changed various parameters. First, since only 11 out of the 12 potential covariates were included in our main model (‘distance to the community’s population center’ was deemed to be redundant with distance to roads and distance to municipality), we tested all other possible combinations of 11 variables. We tested those models with and without spatial lags. We also tested two alternative forest thresholds in 2007 to the one used in our final model. Instead of including all pixels that had at least 10% of their area forested, we tried with 1% and 20%. Finally, we also tested the robustness of our results to a change of definition of our control group. Although in the main model the control group includes all never treated units, and additional units that eventually participate in the treatment but have not participated yet, we also tested models that only included the cells from never treated communities in the control group.

### Robustness to the parallel trend assumption

Generalized DiD leads to unbiased estimates only if the so-called parallel trend assumption that both enrolled and unenrolled sites would have followed the same deforestation trend, conditional on observed covariates, holds. This assumption is inherently untestable but a way to assess its plausibility is to explore how robust the main findings are to increasing deviations from the parallel trend assumption^46^. For each estimate, this method computes the Mbar value that indicates the magnitude of the difference between pre-treatment trends and post-treatment trends. The higher the value, the more confident we can be that our results are significant. For example, if Mbar is equal to 1.5, it means that the difference between pre-trends and post-trends is so important that even if the parallel trend assumption was violated by 1.5 times the magnitude of the largest variation observed in the pre-treatment trends, the results would still be significant. As recommended, we used 1 as a threshold. If Mbar is higher than 1, the treatment effect is significant. Otherwise, the effect may not be robust.

### Checking for intra-community leakage

To determine whether PES contracts partially shifted deforestation to other parts of the community, we used the same identification strategy to assess the effect of the program on the non-enrolled forests of participating communities. A negative, significant estimate would indicate leakage (see Supplementary Note 2).

### Software

Maps were developed using the *R Project for Statistical Computing*, version 4.3.0 and estimates and graphs using *R package DiD*, version 2.1.1 (https://bcallaway11.github.io/did/index.html). Robust analytical standard errors were clustered at the community level in each specification. For our robustness checks, we used the *spec_chart* R package (https://github.com/ArielOrtizBobea/spec_chart) to obtain specification charts. Finally, we used the *honestdid* R package (https://github.com/asheshrambachan/HonestDiD) and the wrap-up used by Pedro Sant’ Anna (https://github.com/pedrohcgs/CS_RR) to obtain the M bar values.

### Limitations

We identify at least three limitations in our methodological approach. First, like all quasi-experimental studies, we have addressed selection bias on observable variables only, and not on unobservable ones, such as pro-conservation behavior, or collective action. We have assumed that the confounding variables adjust for most of them on average. Second, our sample size and unit of analysis do not allow us to quantify the different mechanisms through which cumulative avoided deforestation is induced, but we have shown that spatial targeting sites with deforestation risk is a key enabling condition. Finally, even with the doubly robust estimation, the covariate sets chosen might not capture all the socio-ecological factors that could have an impact on both selection into treatment and outcome^47^, particularly given the complexity of local tenure arrangements, the evolving drivers of forest loss, and the way each PES contract is implemented, which can involve a distinct tertiary organization providing contrasting levels of technical assistance.

### Supplementary Information

Supplementary Information.

## Data Availability

The data that support the findings of this study will be made available upon request to the corresponding author, as well as made accessible through the datasets open repository of the Universitat Autònoma de Barcelona (https://ddd.uab.cat/collection/datasets?ln=en).
