# Author Correction: Sustained participation in a Payments for Ecosystem Services program reduces deforestation in a Mexican agricultural frontier

**DOI:** 10.1038/s41598-024-52284-0

**Published:** 2024-01-18

**Authors:** Hugo Charoud, Sebastien Costedoat, Santiago Izquierdo‑Tort, Lina Moros, Sergio Villamayor‑Tomás, Miguel Ángel Castillo‑Santiago, Sven Wunder, Esteve Corbera

**Affiliations:** 1 Institute of Environmental Science and Technology, Universitat Autònoma de Barcelona, 08193 Bellaterra, Spain; 2 Conservation International, Arlington, VA USA; 3 Instituto de Investigaciones Económicas, Universidad Nacional Autónoma de México, Circuito Mario de La Cueva Ciudad Universitaria, 04510 Mexico City, Mexico; 4 Universidad de los Andes, School of Management, Calle 21 # 1‑20, Bogotá, Colombia; 5 Ostrom Workshop, Indiana University, Bloomington, IN 47408 USA; 6 Departamento de Observación y Estudio de la Tierra, la Atmósfera y el Océano, El Colegio de la Frontera Sur, 29290 San Cristóbal de las Casas, Mexico; 7 European Forest Institute, St. Antoni M. Claret 167, 08025 Barcelona, Spain; 8 Institució Catalana de Recerca i Estudis Avançats (ICREA), Psg. Lluís Companys 23, 08010 Barcelona, Spain; 9 Department of Geography, Universitat Autònoma de Barcelona, 08193 Bellaterra, Spain; 10 Center for International Forestry Research (CIFOR), La Molina, Lima 12, Peru

Correction to: *Scientific Reports* 10.1038/s41598-023-49725-7, published online 15 December 2023

The Supplementary Information file published with this Article contained an error where the caption of Supplementary Figure 7 was incorrect.

This error has now been corrected in the Supplementary Information file that accompanies the original Article.

