# Supplementary material for: Sustained participation in a Payments for Ecosystem Services program reduces deforestation in a Mexican agricultural frontier
Source: Sci Rep. 2023 Dec 15;13:22314. doi: 10.1038/s41598-023-49725-7 (PMC10724165; doi:10.1038/s41598-023-49725-7)
Supplement: Supplementary file 1 — Supplementary Information. [file 41598_2023_49725_MOESM1_ESM.pdf]

Sustained participation in a Payments for Ecosystem Services program reduces deforestation in a Mexican agricultural frontier

Hugo Charoud[1], Sebastien Costedoat[2], Santiago Izquierdo-Tort[3], Lina Moros[4], Sergio Villamayor-Tomás[1,5], Miguel Ángel Castillo-Santiago[6], Sven Wunder[7,10], Esteve Corbera[1,8,9,*]

1. Institute of Environmental Science and Technology, Universitat Autònoma de Barcelona, 08193 Bellaterra, Spain.
2. Conservation International, Arlington, Virginia, United States.
3. Instituto de Investigaciones Económicas, Universidad Nacional Autónoma de México, Circuito Mario de La Cueva Ciudad Universitaria, Mexico City 04510, Mexico.
4. Universidad de los Andes, School of Management, Calle 21 # 1-20, Bogotá, Colombia.
5. Ostrom Workshop, Indiana University, 47408, Indiana, US
6. Departamento de Observación y Estudio de la Tierra, la Atmósfera y el Océano, El Colegio de la Frontera Sur, San Cristóbal de las Casas 29290, Mexico.
7. European Forest Institute, St. Antoni M. Claret 167, ES-08025 Barcelona, Spain.
8. Institució Catalana de Recerca i Estudis Avançats (ICREA), Psg. Lluís Companys 23, 08010 Barcelona, Spain.
9. Department of Geography, Universitat Autònoma de Barcelona, 08193 Bellaterra, Spain.
10. Center for International Forestry Research (CIFOR), La Molina, Lima 12, Peru.

SUPPLEMENTARY MATERIAL

Supplementary Notes 1: Disaggregating short-term contracts between non-renewed contracts and recent contracts.

To understand more precisely the effect of non-renewed contracts, we split the sample in two groups:

- Parcels that entered recently (after 2013) which contract was therefore not renewed yet in 2018 (recent)
- Parcels enrolled that were excluded from PES when they pursued contract renewal (non-renewed)

We kept parcels enrolled in long term-contracts (renewed) as a comparison group and we focused only on Marqués de Comillas (MdC) and Benemérito de las Américas (BdA) because it is in these municipalities where most of the avoided deforestation has happened. Including Maravilla Tenejapa (MT) in the analysis would have only added noise to our estimations.

As shown in Supplementary Table 3, the covariates reveal that non renewed parcels had way lower forest cover rates, indicating that some were included by mistake (technical error) and therefore non-renewed because they should never have been eligible in the first place.

As Supplementary Figure 7 shows, there are two trends among parcels enrolled for 5 years or more. If we distinguish between parcels that were first enrolled and not renewed at the end of those 5 years from parcels that entered PES recently and therefore that did not have to be renewed yet by 2018, we obtain different estimations. In recently enrolled parcels, the magnitude of the effect is similar as the one in parcels enrolled for more than 10 years (0.11, CI[-0.51,0.81]). On the contrary, in the other group, the coefficient appears to be null (0.03, CI[-0.45,0.39]). However, because of low statistical power, those two results are not statistically significant neither from each other nor from 0. We lack long-term data to study fully the more recent PES cohorts.

Supplementary Notes 2: Defining community boundaries.

The boundaries of many communities in the three municipalities studied are still not fully legally recognized, notably due to mismatches between land use change regulations and *de facto* land management, and a limited participation in land titling programs. Therefore, a unified public georeferenced database of *ejidos* boundaries is not available. Nevertheless, we can approximate the *de facto* boundaries of each community by combing different sources of information. First, the main source of information we had was the National Agrarian Registry (RAN) (**"perimetrales"** database), which contained 62 communities (35 in MdC and BdA and 27 in MT). We completed this information with a less recent database (**"ine_2005**" database), produced by the National Institute of Statistics and Geography (INEGI) before 2005 that included more communities in MT (33). However, this database being older, the frontiers are less accurate and to be used with precaution. Finally, and for Mdc and BdA only, we also had access to the community limits used by Costedoat et al. in their 2014 article (**"PlosOne"** database). It contained two more communities than the RAN database and all the land of municipalities was attributed.

We combined this georeferenced information with other sources to improve the accuracy of our database. We first ensured that each community contained a georeferenced village of the same name than the community within its boundary. The georeferenced censuses indeed provided GPS coordinates of each censed village. Since many ejido bear the name of their most important town, we assumed that villages that had the name of a given community belonged to it. We also used physical characteristics such as rivers, roads, or land use change to identify community boundaries. Indeed, when community limits were not clear, but a natural limit was present, the latter was assumed to be also the community border. Finally, PES polygons provided by CONAFOR also suggested information on communities' limits by showing areas of land enrolled by PES contracts. Knowing that every contract was signed by a community, we could infer that land enrolled in a contract under a certain community name belonged to the latter. However, these polygons were less reliable before 2009 when the use of GPS and GIS technologies was not well mastered by the individuals collecting and processing these data.

Given the considerations noted above, the task of defining community boundaries was quite straightforward in MdC and BdA and more challenging in MT. The final database represents our best efforts to delimit community boundaries, and the location of PES polygons within enrolled *ejidos*. To limit the risk of measurement errors, we did not use non-PES plots within enrolled ejidos in our control group.

Supplementary Notes 3: Testing for leakage at community level.

PES have been shown to sometimes shift deforestation to unenrolled parcels instead of avoiding deforestation. In this regard, the positive effect we find could be due to this leakage

effect and not to a real effect of the program. To ensure the effect we measure is avoided deforestation and not displaced deforestation, we tested for leakage.

Methods: Community level leakage would mean that unenrolled forested areas of participating communities would have, *ceteris paribus*, higher deforestation rates than forested areas of non-participating communities. To test this hypothesis, we compare every forested grid cell that belongs to a community that has signed a PES contract but is not under PES with all forested pixels that are in non-participating or not yet participating communities. We then apply the same estimation strategy as for the main results: doubly robust CSA estimator with the same confounding variables as covariates.

Results: We find no significant effect of leakage on the long run. The effect is negative but very low compared to the effect we measured in terms of avoided deforestation. If this leakage effect exists, it is not of the same magnitude as the PES effect on deforestation we measure. This indicates that even if leakage takes place, PES contracts are still effective.

Limitations: This identification strategy is however not perfect. Indeed, since we only have access to community boundaries, we are less precise than if we had access to individual property limits. We therefore have low statistical power. Moreover, this strategy only considers on-site leakage and no other types of leakage that might also have a significant (positive or negative) effect.

Supplementary Notes 4: Reclassification of forest cover measures.

Our outcomes of interest are forest cover loss and forest degradation. We used Vancutsem et al (2021) Tropical Moist Forests (TMF) product in the version covering 1990 to 2020 (European Commission Joint Research Centre-JRC) to compute our outcome variables. The JRC database maps the undisturbed tropical moist forest in 1990 and all the following years. Then, for each pixel considered as forested at the baseline year, the absence or presence of a disturbance is measured every year. If any disturbance is noticed, the pixel is either classified as degraded, or as deforested. Selective logging, fires, and unusual weather events (hurricane, drought, blowdown) are considered as forest degradation. Degradation is defined as a loss of productivity, a loss of biodiversity and a reduction of carbon storage. Degraded pixels can later eventually be deforested. However, if the disturbance disappears, then the pixel is classified as “Tropical moist forest regrowth”. This represents the fact that a parcel of forest that has been deforested will not over a short-term period recover the level of biodiversity or carbon storage it had. We reprocessed the data to remove the regrowth category, because observation in the field in southern Chiapas showed that what is classified as regrowth in global remote sensing product often corresponds to conversion to oil palm or rubber plantations. Therefore, in our database, regrowth pixels are always classified as degraded forests.

Our map then considers three types of forest cover change (See Supplementary Figure 1):

A. Direct deforestation: from undisturbed forest cover to deforested area.
B. Deforestation after degradation: from degraded forest to deforested area.
C. Forest degradation: degradation of undisturbed forest cover.

In our research, we use only two outcome variables. The first one captures only the annual loss of undisturbed forest cover, either due to deforestation (A) or degradation (C). Our second outcome only takes into account degradation (C), and more specifically the cumulated sum of total forest degradation since baseline year.

Supplementary Notes 5: Disaggregating the effect at the cohort level.

We disaggregated the effect of PES for each cohort to assess the effect of different payment levels, since PESL contracts offering higher payments were implemented from 2010 onwards (see Supplementary Figure 2). As shown in Supplementary Figure 15, all the PES cohorts except for 2009 have a significant positive effect on deforestation. However, the variation of this effectiveness does not follow the change in payments. This does not necessarily suggest that offering higher payments had not led to more avoided deforestation but instead that there are also other factors at play when it comes to participating in PES, such as the level of trust with the program, the information received at community level, etc.

SUPPLEMENTARY FIGURES

Supplementary Fig. 1: Annual forest cover change (ha) by deforestation and degradation status in Maravilla Tenejapa (MT), Marqués de Comillas (MdC) and Benemérito de las Américas (BdA), Chiapas, Mexico (source: JRC database).

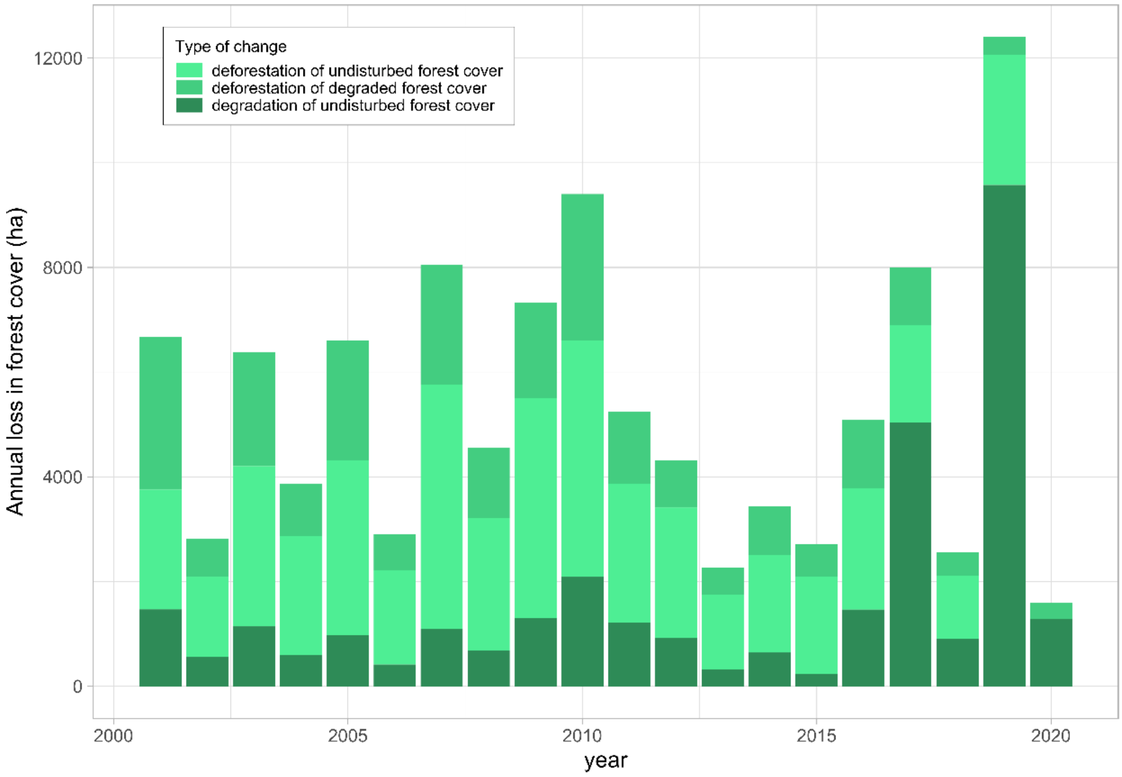

Caption: Forest degradation has become the main driver of forest cover change since 2016 in the studied municipalities, whereas deforestation of primary forest cover was dominating during the previous years.

Supplementary Fig. 2: Number of contracts by type (starting year between 2008 and 2018) in MT, MdC and BdA, Chiapas, Mexico.

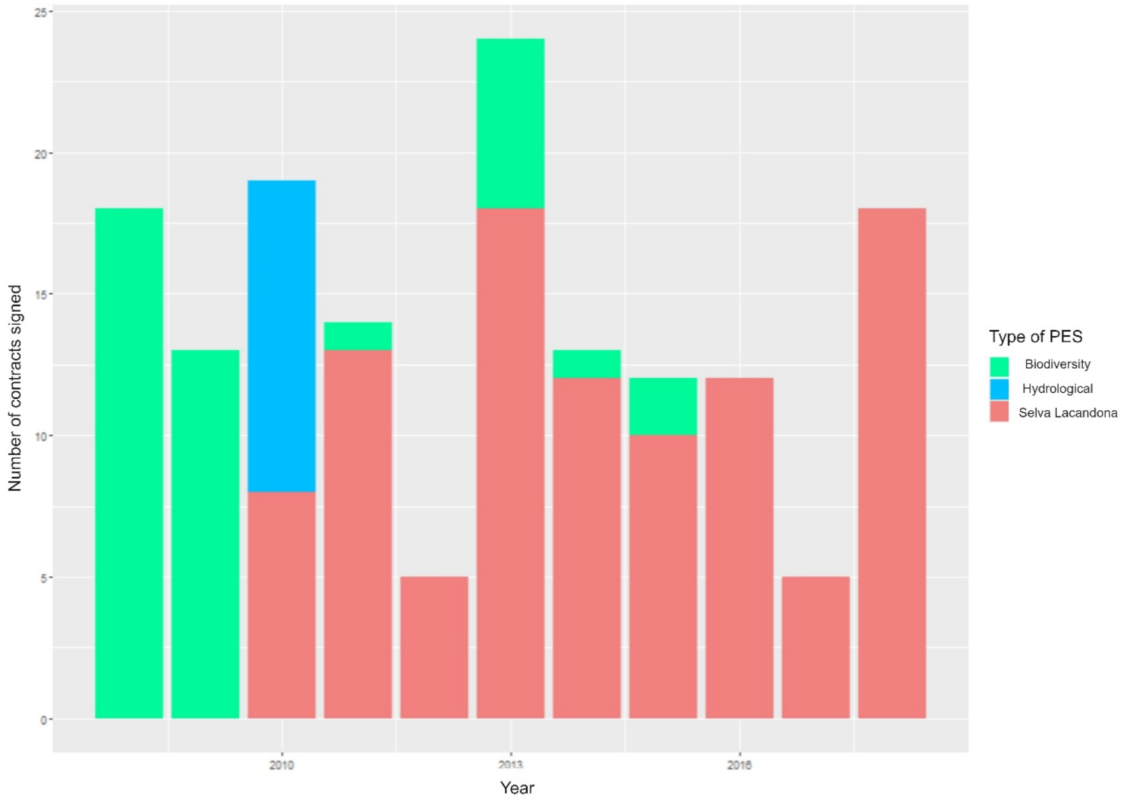

Caption: PESL became the main type of PES contract implemented in the region from 2010 onwards. This contract offered a notably higher payment by hectare than the formerly offered modalities. After 2014, all PES modalities obliged participants to invest a share of payments (30%- 50%) in specific conservation activities rather than distributing such revenue among participants.

Supplementary Fig. 3: Enrolment in PES at community (*ejido*) level.

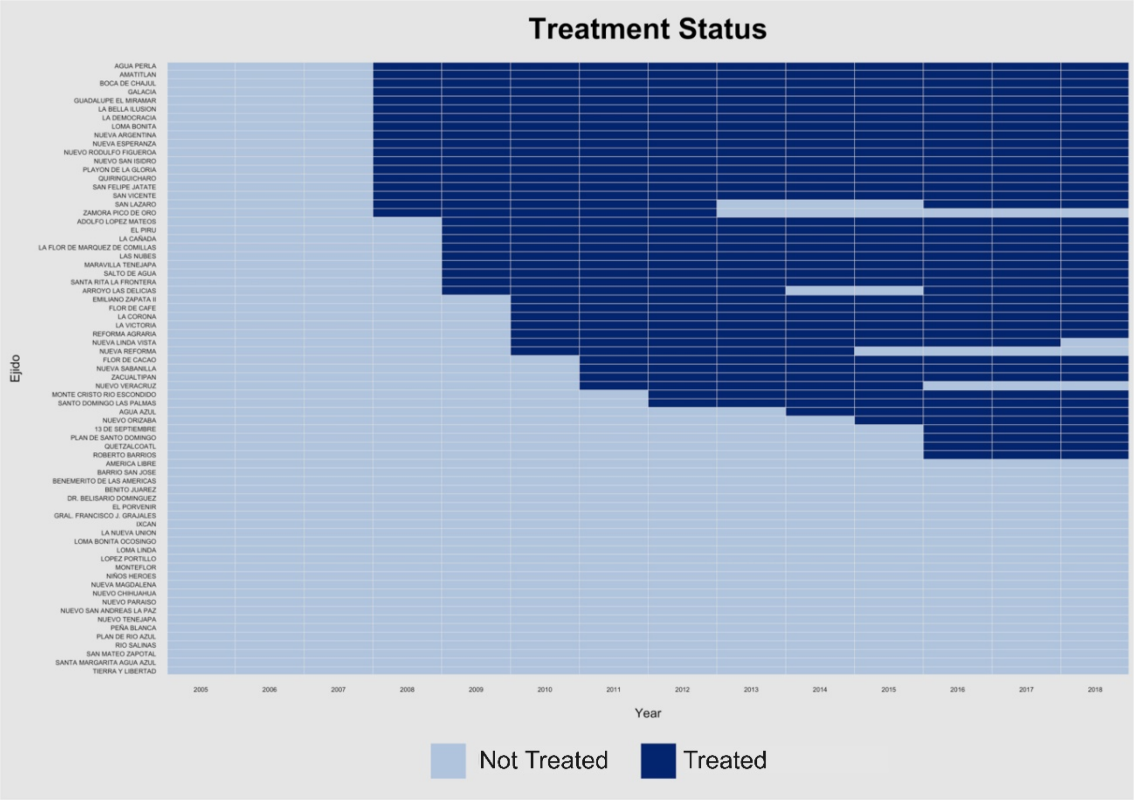

Caption: The first PES contract in the region was signed in 2008. Most (but not all) *ejido*s that have enrolled in PES renew their contracts after 5 years.

Supplementary Fig. 4: Annualized deforestation rates in the three regions between 2000 and 2007 in treated and untreated areas.

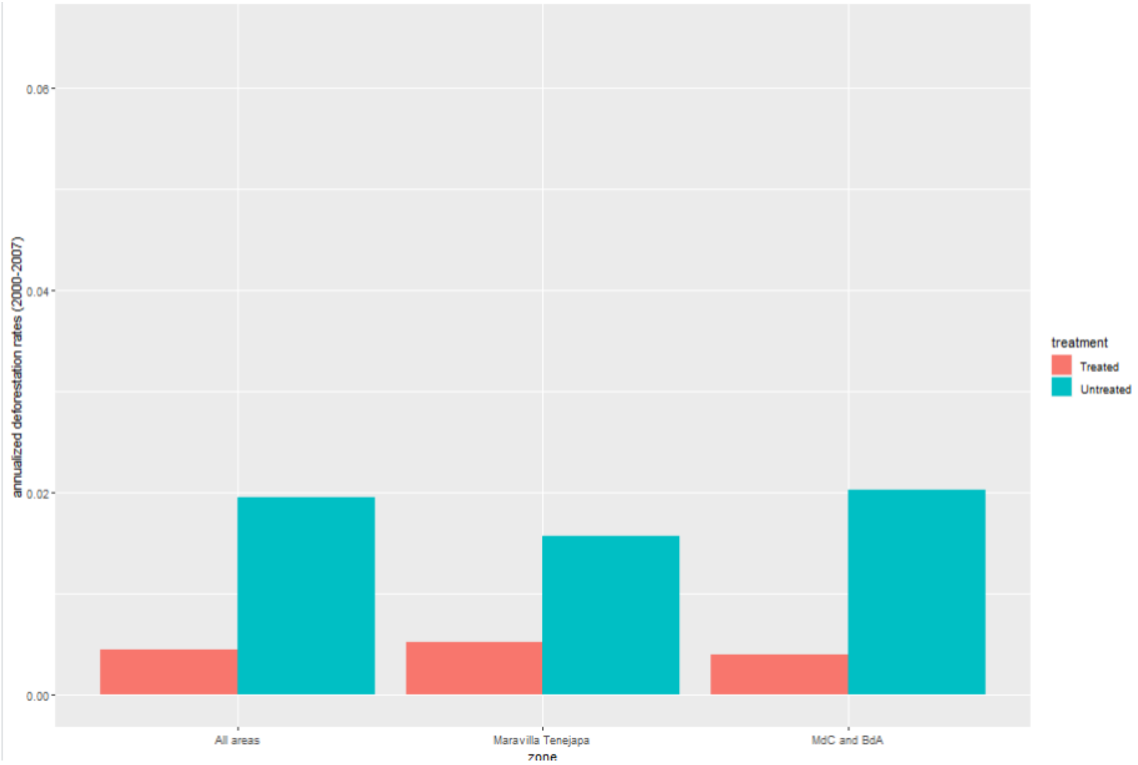

Caption: Before the beginning of PES contracts, deforestation was lower in areas that would later be enrolled in a PES contract.

Supplementary Fig. 5: Annualized deforestation rates in the three regions between 2008 and 2020 in treated and untreated areas.

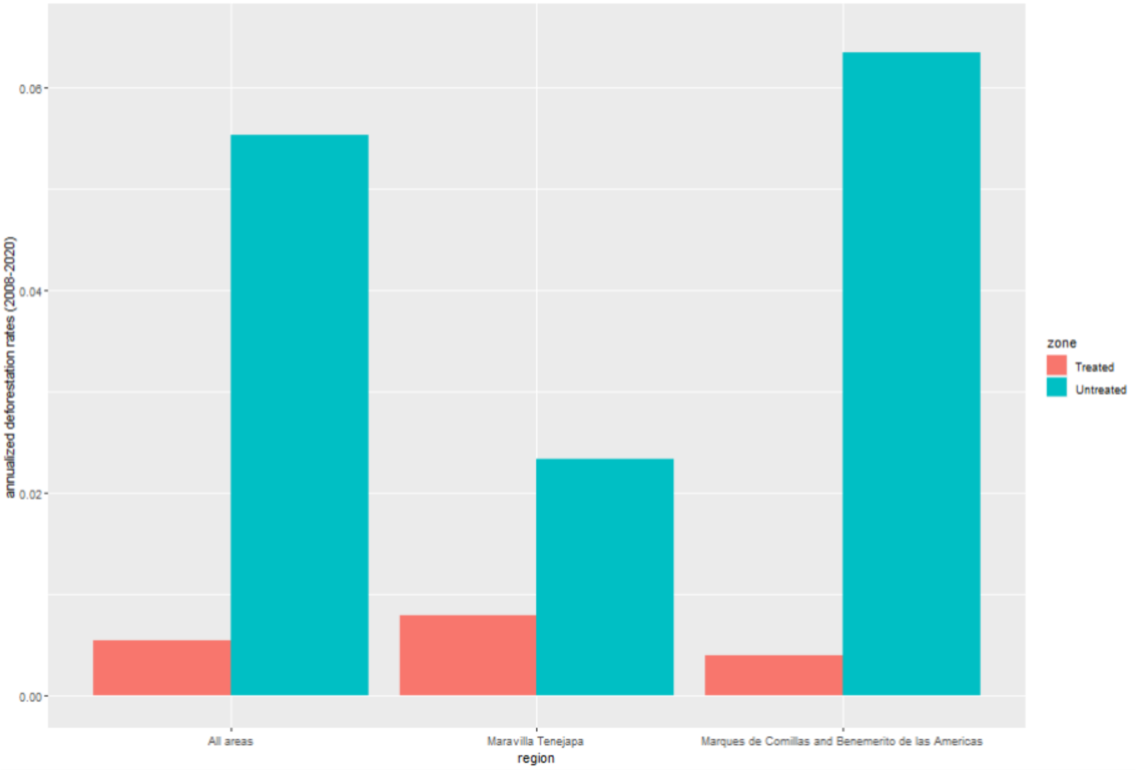

Caption: During PES contracts deforestation remained low in enrolled areas but was very important in unenrolled parcels.

Supplementary Fig. 6: Event study for leakage effect on deforestation.

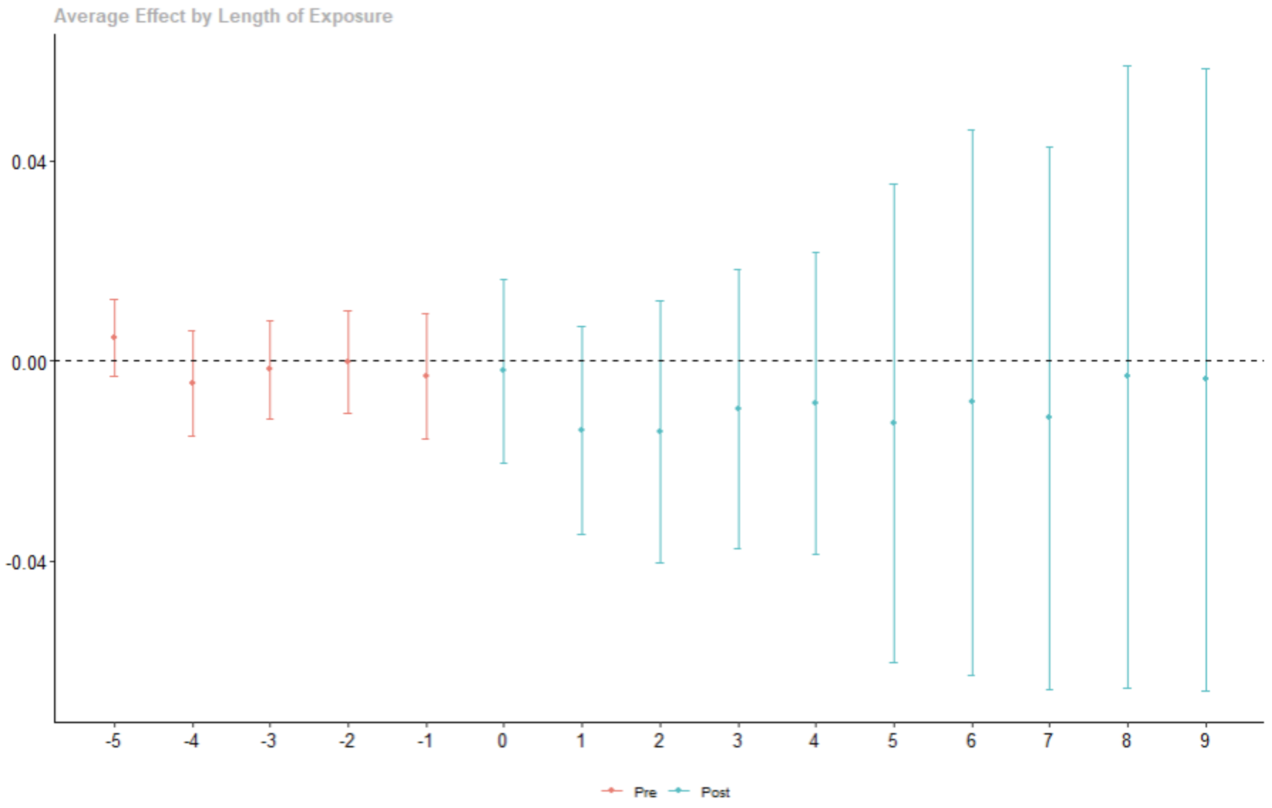

Caption: At community level, we cannot reject the null hypothesis that there is no leakage effect of enrolment in PES.

Supplementary Fig. 7: Treatment effect of PES for each type of contract in MdC and BdA, Chiapas, Mexico.

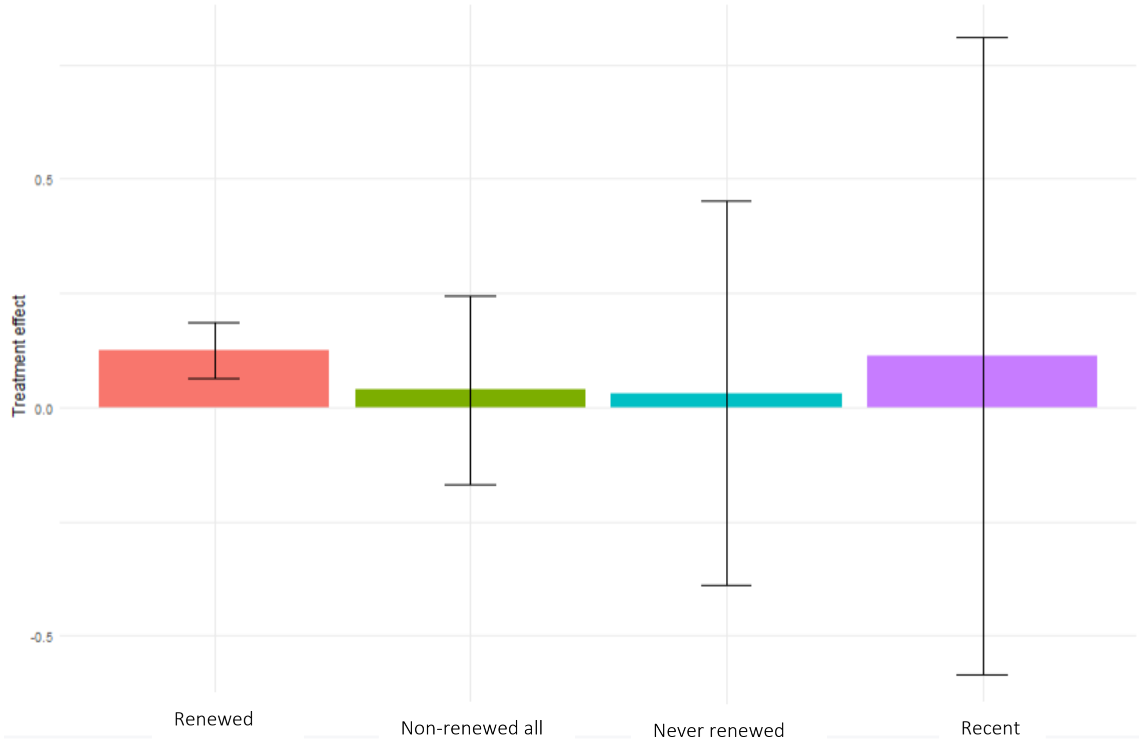

Caption: The ‘Renewed’ category represents contracts that have been renewed once or twice. The ‘Non-renewed all’ category represents contracts that at some point were not renewed (but may have been renewed once and subsequently not-renewed). The ‘Never renewed’ category represents contracts that were not renewed once the first contract expired. The ‘Recent’ represents contracts signed too recently to have been signed yet. Recent contracts seem to be as effective as renewed contract whereas the “never renewed” seem to show a lower effect. However, given our sample size, we cannot reject the null hypothesis.

Supplementary Fig. 8: Robustness checks: effect of renewed contracts on deforestation in the whole study area.

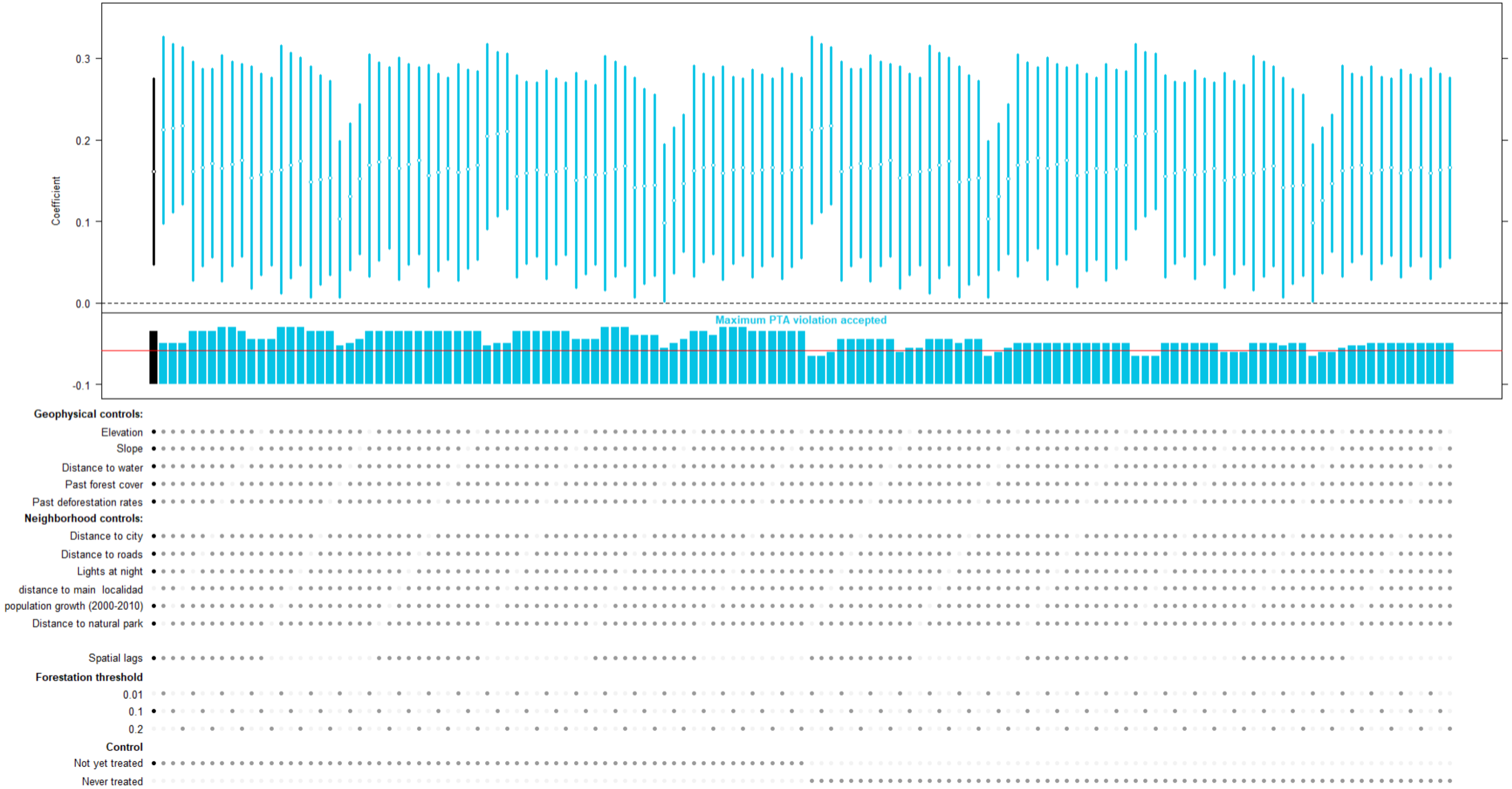

Caption: Mbar values were computed for each specification. Out of all the 133 models all of them allow to reject the null hypothesis at the 5% threshold. Moreover, half of the model's results are robust to a parallel trend violation higher than the maximum violation observed in the pre-trends.

Supplementary Fig. 9: Robustness checks - effect renewed contracts on deforestation in MT, MdC and BdA, Chiapas, Mexico.

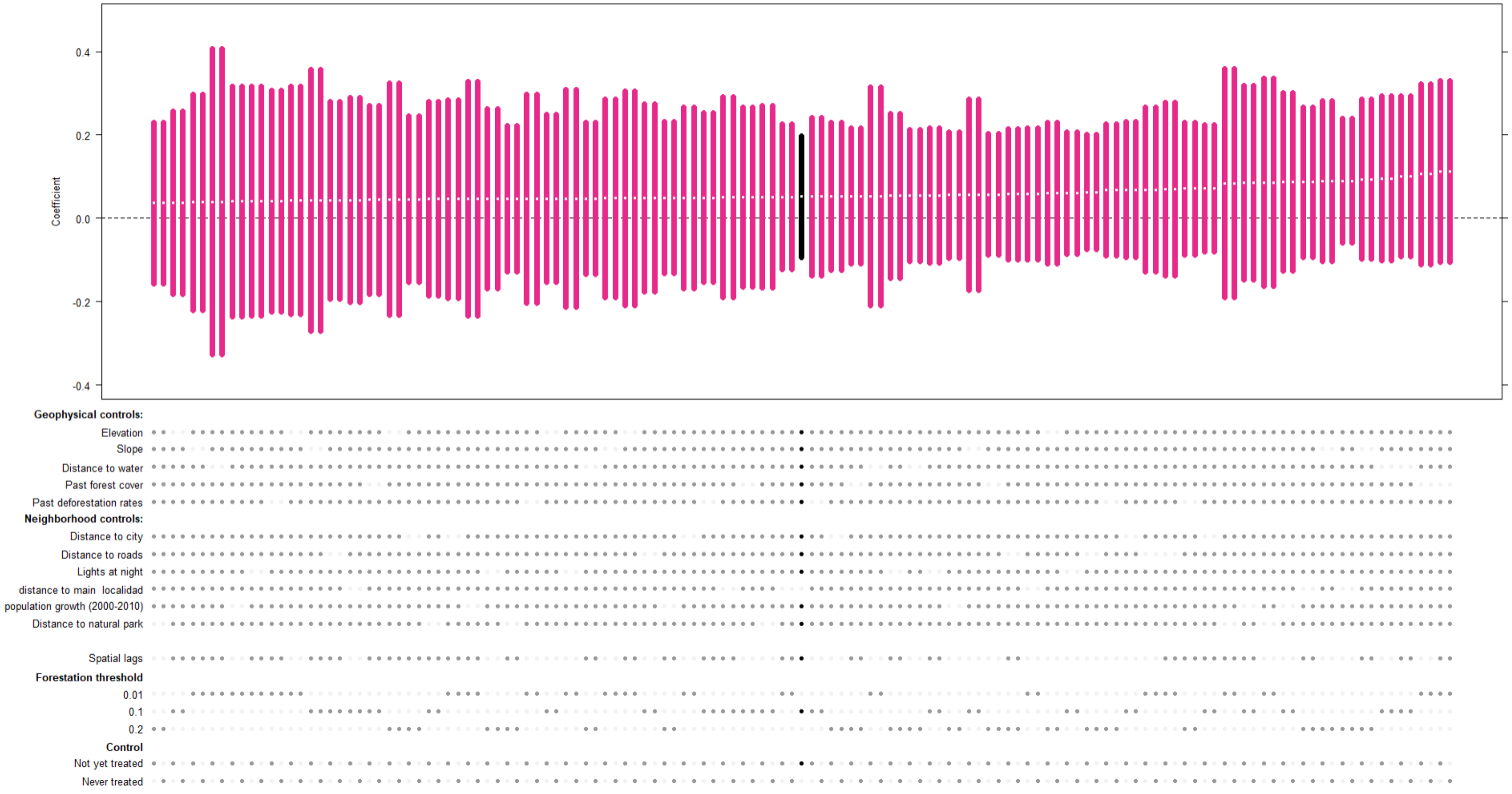

Caption: Out of all the 133 models none of them allow to reject the null hypothesis at the 5% threshold.

Supplementary Fig. 10: Robustness checks - effect of renewed contracts on deforestation in MdC and BdA, Chiapas, Mexico.

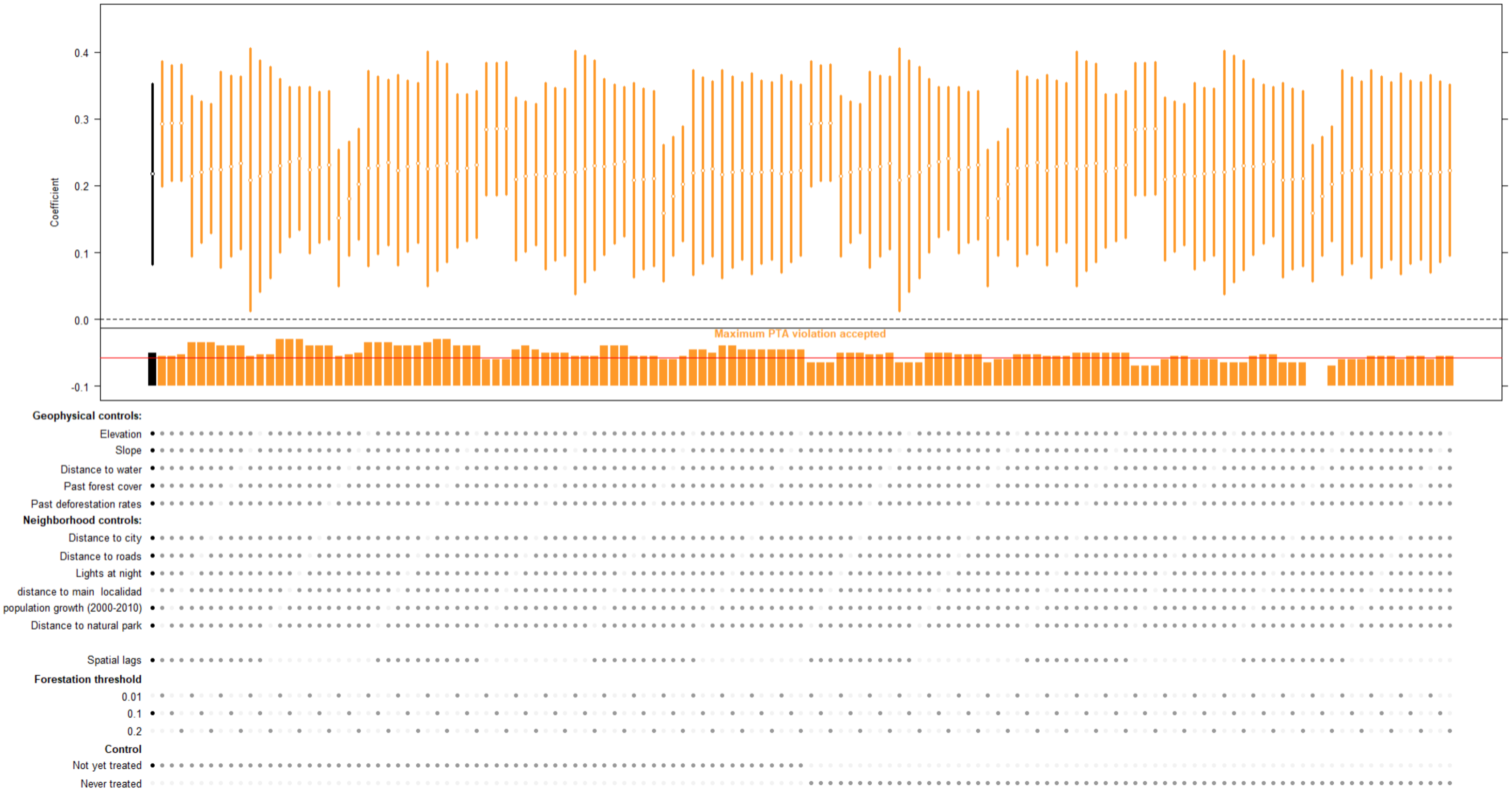

Caption: Out of all the 133 models all of them allow to reject the null hypothesis at the 5% threshold. Moreover, half of the model's results are robust to a parallel trend violation higher than the maximum violation observed in the pre-trends.

Supplementary Fig. 11: Robustness checks: effect of renewed contracts on forest degradation in MT, MdC and BdA, Chiapas, Mexico.

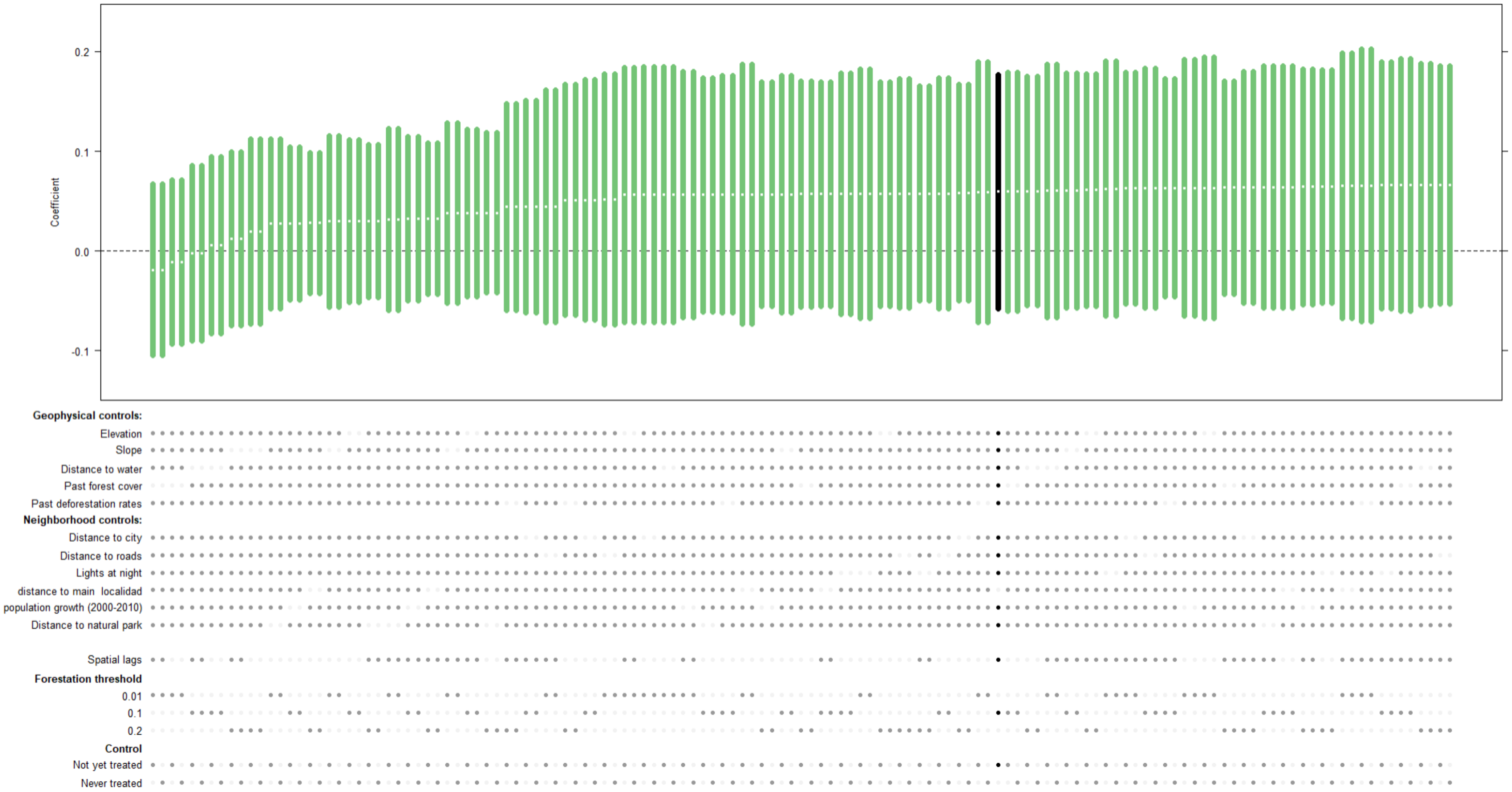

Caption: Out of all the 133 models none of them allow to reject the null hypothesis at the 5% threshold.

Supplementary Fig. 12: Number of PES contracts signed, and area enrolled in PES between 2008 and 2018 in MT, MdC and BdA, Chiapas, Mexico.

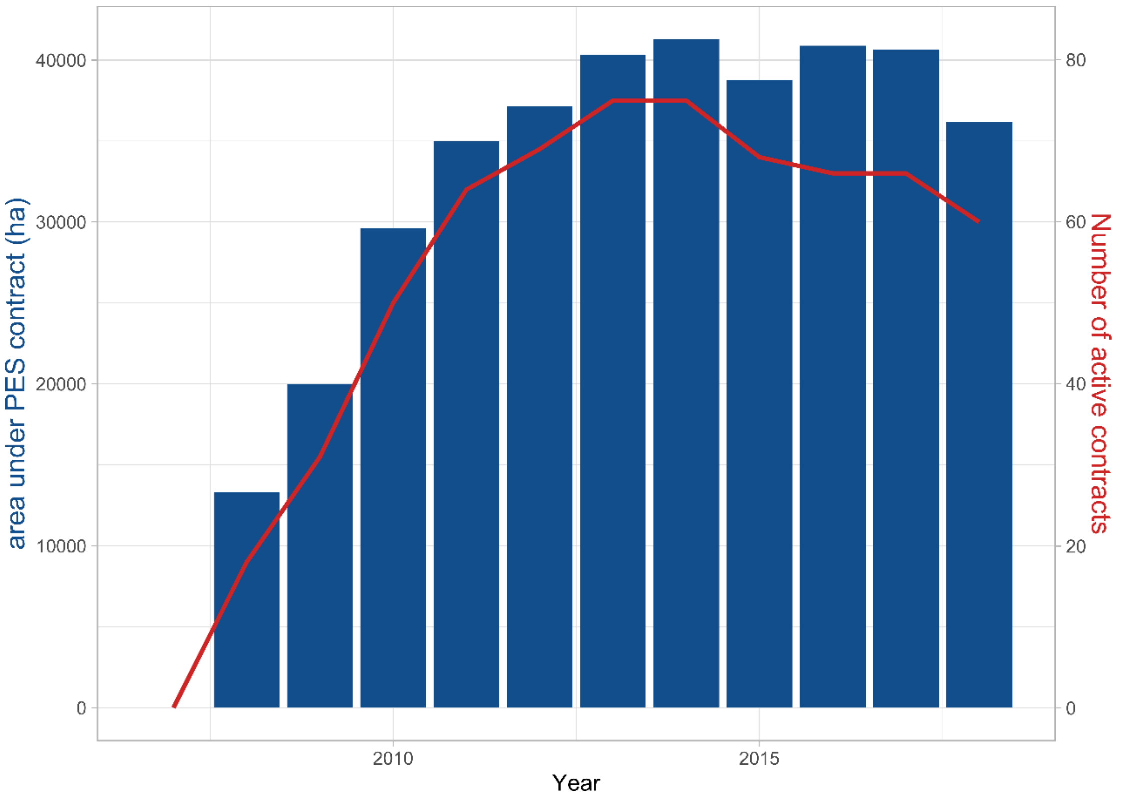

Caption: Both PES coverage in hectare and number of contracts have increased between 2008 and 2013, and slowly decreased after 2013.

Supplementary Fig. 13: Disaggregated panel DiD for renewed contracts and avoided deforestation as outcome.

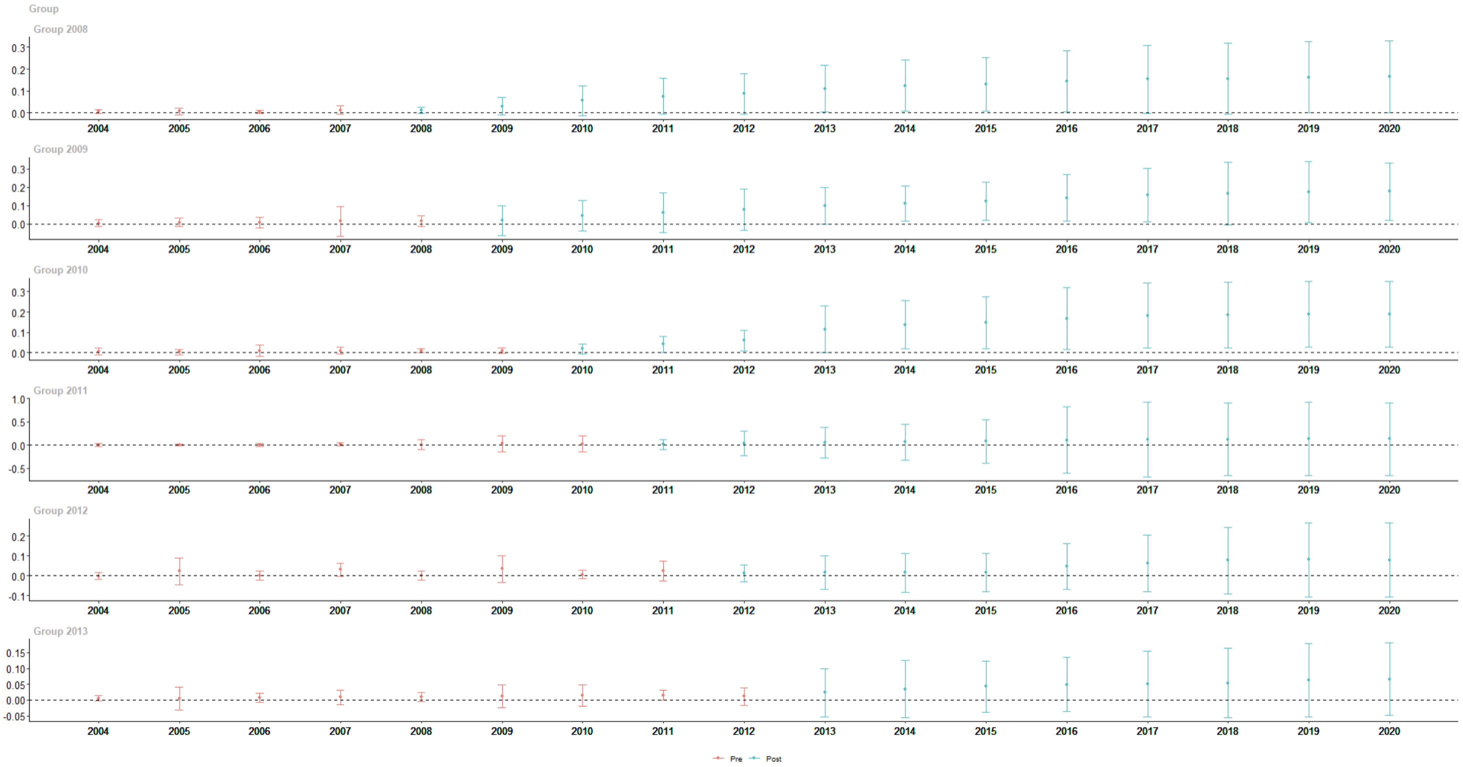

Caption: By disaggregating by cohort, it can be observed that the early cohorts have a significant impact whereas it is not possible to reject the null hypothesis for more recent cohorts.

Supplementary Fig. 14: Event study graph for forest degradation for renewed contracts in MT, MdC and BdA, Chiapas, Mexico.

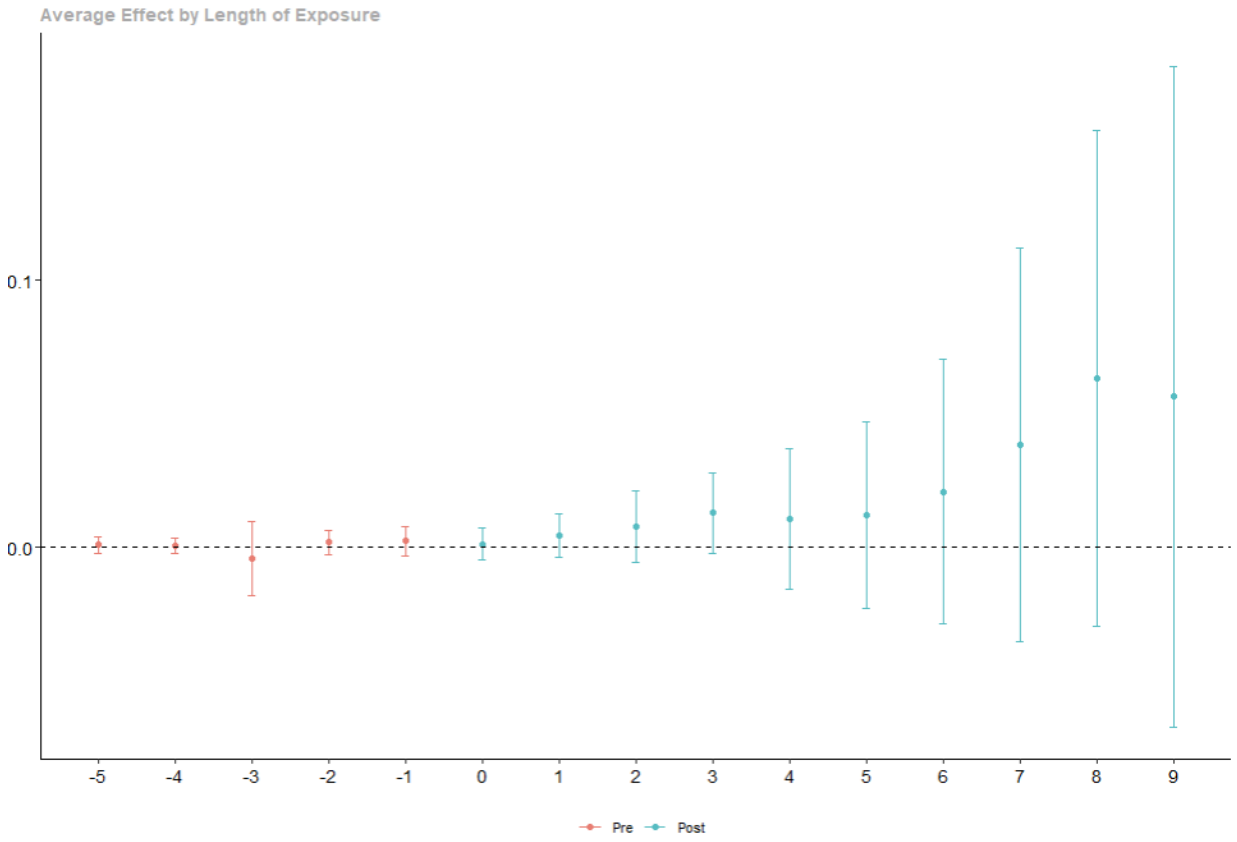

Caption: We cannot reject the hypothesis that renewed PES contracts did not have an impact on forest degradation over the 10-year contracting period.

Supplementary Fig. 15: Effect of PES on deforestation for renewed contracts in each cohort in MdC and BdA, Chiapas, Mexico.

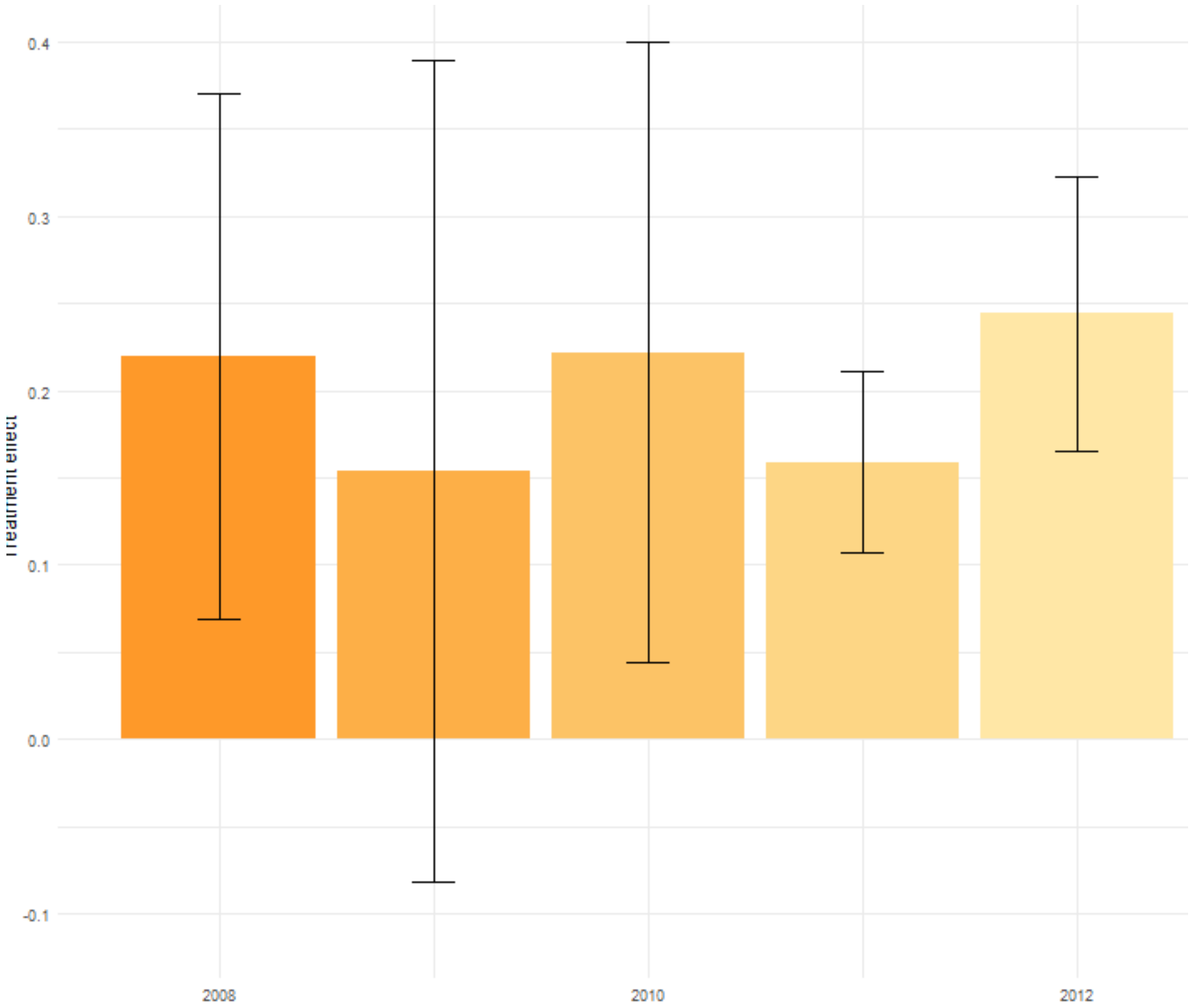

Caption: By restricting the sample to BdA and MdC and disaggregating by cohorts, it can be observed that each cohort of renewed PES had significant impact (except for the 2009 cohort). This exception can be explained by the lower sample size for this cohort. However, we do not find evidence that later cohorts with higher payments led to higher treatment effects. Other factors that we cannot disentangle may explain such lower performance.

SUPPLEMENTARY TABLES

Supplementary Table 1: Geographical heterogeneity of MT, versus MdC and BdA, Chiapas, Mexico.

	Maravilla Tenejapa (MT)	Marqués de Comillas (MdC) and Benemérito de las Américas (BdA)
**Elevation (m)**		
Mean	336.2	158.3
SD	146.7	26.1
Range	133.0 – 1075.0	85.0 – 275.0
**Slope (º)**		
Mean	13.2	5.67
SD	10.5	3.39
Range	0.0- 74.0	0.0 – 39.0
**distance to inland water (m)**		
Mean	2860.3	2316.0
SD	2620.4	1886
Range	0.3 – 10 474.7	0.3 – 9669.0
**Share of forest cover in 2007 (%)**		
	55%	45%
**Annualized deforestation rate (2000-2007)**		
	1.3%	3.1%

Caption: Average of all pixels in each sub-region. MT is mountainous, so agricultural expansion is difficult; MdC and BdA are mostly flat, crosscut by local rivers, with soils more suited for agricultural conversion.

Supplementary Table 2: Full covariate table for treated and untreated areas in MT, and MdC and BdA, Chiapas, Mexico.

	**MT**		**MdC and BdA**	
	enrolled areas	unenrolled areas	enrolled areas	unenrolled areas.
**Elevation (m)**				
Mean	410.183	356.222	169.017	160.615
SD	145.317	191.015	21.334	27.614
Range	148.129 - 913.985	143.054 - 1064.655	103.678 - 255.163	101.716 - 247.715

	MT		MdC and BdA	
	enrolled areas	unenrolled areas	enrolled areas	unenrolled areas.
**Slope (º)**				
Mean	16.775	15.365	6.308	6.097
SD	9.569	9.424	2.285	2.206
Range	0.924 - 59.502	1.228 - 60.653	0.000 - 28.247	0.000 - 25.334
**forest cover share in 2007**				
	0.8580	0.3124	0.9368	0.3677
**Annualized deforestation rate (2000-2008) (%)**				
	0.521	0.521	0.405	0.405
**distance to inland water (m)**				
Mean	3369.713	2504.853	2528.244	2541.893
SD	2475.602	2538.546	1989.268	1698.673
Range	8.069 - 10520.922	3.999 - 9981.036	3.177 - 9608.604	5.754 - 9646.006
**distance to roads (m)**				
Mean	932.878	1263.526	2090.954	1868.145
SD	667.598	973.788	1426.276	1148.854
Range	14.033 - 3636.439	13.885 - 4838.042	22.425 - 7358.678	22.355 - 5947.634
**distance to main city of the municipality**				
Mean	10627.206	15710.420	21259.787	19288.801
SD	6114.205	6898.457	9976.747	5719.545
Range	196.215 - 30517.433	4258.070 - 35753.190	441.723 - 44430.229	798.753 - 34141.040
**distance to protected area (m)**				
Mean	4953.696	3266.635	16825.774	20073.854
SD	4253.691	3414.882	12162.786	7236.232
Range	0.000 - 16294.831	0.000 - 13960.378	0.000 - 44243.724	1633.068 - 40245.510
**lights at night (2014) (lux)**				
Mean	5.088	5.300	4.178	4.412
SD	2.409	1.922	2.888	2.731
Range	0.000 - 8.000	0.000 - 7.000	0.000 - 8.000	0.000 - 11.000
**demographic growth rate (2000-2010)**				
Mean	0.179	0.097	0.140	0.196

	MT		MdC and BdA	
	enrolled areas	unenrolled areas	enrolled areas	unenrolled areas.
SD	0.286	0.256	0.192	0.237
Range	-0.331 - 1.000	-0.713 - 0.439	-0.144 - 0.793	-0.309 - 1.000

Supplementary Table 3: Summary statistics of confounders for renewed parcels, non-renewed, parcels and recent parcels.

	recent	Non-renewed	renewed
**Elevation (m)**			
Mean	170.357	168.490	178.450
SD	19.530	17.804	21.773
Range	117.192 - 241.380	114.161 - 231.070	111.407 - 255.163
**Slope (º)**			
Mean	6.328	6.212	6.688
SD	2.400	2.113	2.511
Range	1.777 - 26.724	1.660 - 22.375	1.477 - 28.247
**forest cover share in 2007**			
Mean	0.940	0.883	0.960
SD	0.156	0.229	0.128
Range	0.008 - 1.000	0.000 - 1.000	0.000 - 1.000
**distance to inland water (m)**			
Mean	2598.512	2488.735	2263.245
SD	1813.833	2014.400	1492.480
Range	14.679 - 9142.858	9.652 - 9477.571	12.243 - 6904.824
**distance to roads (m)**			
Mean	2260.478	2138.308	2121.007
SD	1324.232	1364.573	1366.847
Range	25.376 - 6554.332	23.219 - 6337.784	24.957 - 6310.307
**distance to main city of the municipality**			
Mean	26615.152	20930.392	18994.062
SD	9580.025	11239.868	8169.855
Range	5983.354 - 40992.305	3314.762 - 40212.490	4949.121 - 37534.880
**distance to protected area (m)**			
Mean	24743.715	16853.271	9814.805
SD	11388.321	12771.183	8599.877
Range	283.709 - 40841.450	189.993 - 39980.930	180.540 - 37409.020
**night_lights_2014**			
Mean	4.564	4.182	3.675
SD	2.702	2.976	2.956
Range	0.000 - 7.000	0.000 - 7.000	0.000 - 7.000
**growth_baseline**			
Mean	0.208	0.137	0.151
SD	0.203	0.178	0.230

	recent	Non-renewed	renewed
Range	-0.144 - 0.793	-0.144 - 0.793	-0.144 - 0.793

Supplementary Table 4: Summary statistics for confounders for each cohort of renewed contracts.

	2008	2009	2010	2011	2012
**Elevation (m)**					
Mean	170.870	183.481	182.381	161.107	178.980
SD	20.549	20.773	18.446	19.637	13.354
Range	111.407 - 244.987	122.502 - 255.163	115.698 - 232.665	114.161 - 227.727	154.513 - 217.618
**Slope (º)**					
Mean	6.527	6.986	6.435	6.361	6.196
SD	2.331	2.915	2.254	2.101	1.947
Range	1.477 - 22.375	2.000 - 27.306	1.600 - 24.197	1.725 - 17.144	1.903 - 12.696
**forest cover share in 2007**					
Mean	0.945	0.952	0.950	0.931	0.886
SD	0.162	0.143	0.149	0.176	0.204
Range	0.000 - 1.000	0.008 - 1.000	0.000 - 1.000	0.010 - 1.000	0.002 - 1.000
**distance to inland water (m)**					
Mean	2036.975	1961.116	3182.478	1490.240	3973.400
SD	1325.445	1415.081	2006.349	1094.309	1301.183
Range	16.303 - 5867.373	12.243 - 6080.534	21.878 - 9477.571	24.062 - 5012.765	1538.326 - 6442.926
**distance to roads (m)**					
Mean	2324.465	2093.467	2387.659	1734.373	1581.931
SD	1376.925	1231.556	1519.111	992.685	1485.976
Range	26.721 - 6054.546	23.895 - 6310.307	23.686 - 6259.062	24.959 - 6337.784	24.170 - 6279.831
**distance to main city of the municipality**					
Mean	15831.424	22641.261	15911.542	28082.292	13063.288
SD	7983.038	5751.063	6195.864	10615.466	2998.573
Range	3314.762 - 33639.024	5210.832 - 33226.747	5363.994 - 35405.134	5645.868 - 37846.860	7242.347 - 17897.790
**distance to protected area (m)**					
Mean	7278.844	9831.102	9018.130	26942.337	12395.567

	2008	2009	2010	2011	2012
SD	4546.611	8434.677	5688.173	11595.971	3039.039
Range	243.571 - 15933.060	274.472 - 30543.360	180.540 - 27846.760	259.441 - 37504.400	6425.237 - 17205.630
**night_lights_2014**					
Mean	3.546	3.552	2.835	6.018	5.705
SD	2.917	2.936	2.973	1.543	2.250
Range	0.000 - 7.000	0.000 - 7.000	0.000 - 7.000	0.000 - 7.000	0.000 - 7.000
**growth_baseline**					
Mean	0.026	0.204	0.142	0.224	0.246
SD	0.058	0.316	0.189	0.188	0.044
Range	-0.102 - 0.105	-0.144 - 0.793	-0.135 - 0.449	-0.102 - 0.355	0.222 - 0.325

Supplementary Table 5: Covariates definition and sources.

Variable	Defintion	Source
Distance to Inland water	Euclidean distance (m)	INEGI
Distance to main *ejido* village	Euclidean distance (m) normalized at the ejido level	Censo data
Distance to Municipio center	Euclidean distance (m)	Censo data
Distance to road	Euclidean distance (m)	INEGI
Distance to nearest protected area	Euclidean distance (m)	INEGI
Lights at night	Digital number corresponding to illumination for the year 2014 (0 means no lights, and the highest value 63 is the highest lighting intensity detected by the sensor, often corresponding to dense urban areas).	https://www.nature.com/articles/s41597-020-0510-y
deforestation rate	Rate of depletion of total forest cover (2000-2007)	https://forobs.jrc.ec.europa.eu/TMF/download/
Forest cover	Total forest cover	https://forobs.jrc.ec.europa.eu/TMF/download/
Elevation	In meters	https://cmr.earthdata.nasa.gov/search/concepts/C1575731655-LPDAAC_ECS.html
Slope	In degree	https://cmr.earthdata.nasa.gov/search/concepts/C1575731655-LPDAAC_ECS.html
Population growth	Demographic growth rate between 2000 and 2010 at the ejido level	INEGI census data
